# *Giardia duodenalis* enolase is secreted as monomer during trophozoite-epithelial cell interactions, activates plasminogen and induces necroptotic damage

**DOI:** 10.3389/fcimb.2022.928687

**Published:** 2022-08-25

**Authors:** Elisa Barroeta-Echegaray, Rocío Fonseca-Liñán, Raúl Argüello-García, Rafael Rodríguez-Muñoz, Rosa María Bermúdez-Cruz, Porfirio Nava, M. Guadalupe Ortega-Pierres

**Affiliations:** ^1^ Department of Genetics and Molecular Biology, Centro de Investigación y de Estudios Avanzados del Instituto Politécnico Nacional, Mexico City, Mexico; ^2^ Department of Physiology, Biophysics and Neurosciences, Centro de Investigación y de Estudios Avanzados del Instituto Politécnico Nacional, Mexico City, Mexico

**Keywords:** *Giardia duodenalis*, enolase, plasminogen activation, necroptosis, epithelial cells

## Abstract

Enolase, a multifunctional protein expressed by multiple pathogens activates plasminogen to promote proteolysis on components of the extracellular matrix, an important event in early host-pathogen interactions. A secreted form of enolase that is released upon the interaction of trophozoites with epithelial cells has been detected in the secretome of *G. duodenalis*. However, the role of enolase in the host-pathogen interactions remains largely unknown. In this work, the effects of *G. duodenalis* enolase (Gd-eno) on the epithelial cell model (IEC-6) were analyzed. Firstly, the coding sequence of *Giardia* enolase was cloned and the recombinant protein used to raise antibodies that were then used to define the localization and role of enolase in epithelial cell-trophozoite interactions. Gd-eno was detected in small cytoplasmic vesicles as well as at the surface and is enriched in the region of the ventral disk of *Giardia* trophozoites. Moreover, the blocking of the soluble monomeric form of the enzyme, which is secreted upon interaction with IEC-6 cells by the anti-rGd-eno antibodies, significantly inhibited trophozoite attachment to intestinal IEC-6 cell monolayers. Further, rGd-eno was able to bind human plasminogen (HsPlg) and enhanced plasmin activity *in vitro* when the trophozoites were incubated with the intrinsic plasminogen activators of epithelial cells. In IEC-6 cells, rGd-eno treatment induced a profuse cell damage characterized by copious vacuolization, intercellular separation and detachment from the substrate; this effect was inhibited by either anti-Gd-eno Abs or the plasmin inhibitor ϵ- aminocaproic acid. Lastly, we established that in epithelial cells rGd-eno treatment induced a necroptotic-like process mediated by tumor necrosis factor α (TNF-α) and the apoptosis inducing factor (AIF), but independent of caspase-3. All together, these results suggest that *Giardia* enolase is a secreted moonlighting protein that stimulates a necroptotic-like process in IEC-6 epithelial cells *via* plasminogen activation along to TNFα and AIF activities and must be considered as a virulence factor.

## Introduction

Giardiasis is a parasitic diarrheal disease (Karanis et al., 2007) caused by the protozoan Giardia duodenalis (syn. G. lamblia or G. intestinalis) that is transmitted orally via contaminated drinking water or food. The persistent infection of the gastrointestinal tract by Giardia directly affects nutrient absorption and causes intestinal epithelial damage (Ankarklev et al., 2010; Cotton et al., 2011). The establishment, development and maintenance of Giardia infection are intimately related to the attachment of trophozoites to epithelial cells. Unravelling the molecular basis of this process is important for developing novel strategies aimed at controlling giardiasis.

The interaction of G. duodenalis trophozoites with epithelial cells affects the transcriptome and secretome of the parasite (Ringqvist et al., 2011; Ma’ayeh and Brook-Carter, 2012). For instance, the secretion of cysteine proteinases (Rodríguez-Fuentes et al., 2006), variant surface proteins (VSPs), high-cysteine membrane proteins (HCMPs), arginolytic enzymes, such as arginine deiminase (ADI) and ornithine carbamoyl transferase (OCT), and glycolytic enzymes, including enolase (Gd-eno), are increased in trophozoites which are in contact with epithelial cells (Ringqvist et al., 2008). Some of these molecules are involved in the pathogenesis of giardiasis. In that context, the VSP-type protease, VSP9B10A (Cabrera-Licona et al., 2017) and the cysteine proteinase giardipain-1 (Ortega-Pierres et al., 2018) markedly affect epithelial integrity, and the cysteine proteinases (CP) CP14019 (giardipain-1), CP16779 and CP16160 (Roxström-Lindquist et al., 2005) modulate inflammatory responses in the intestinal mucosa (Liu et al., 2018). In contrast, ADI and OCT, two enzymes of the arginine dihydrolase pathway, accelerate enterocyte turnover (Eckmann et al., 2000; Stadelmann et al., 2013). However, the role(s) of other proteins of the secretome, such as enolase, in the host-pathogen interactions remain(s) largely unknown.

Enolase (EC 4.2.1.11, syn. 2-phosphoglycerate hydrolase) is a conserved, ubiquitous metalloenzyme that catalyzes the reversible conversion of 2-phosphoglycerate (2PGA) into phosphoenolpyruvate (PEP) during glycolysis, but can also be a multifunctional protein (Day et al., 1993; Pancholi, 2001; Díaz-Ramos et al., 2012). Gd-eno is one of the most abundantly expressed enzymes during the Giardia life cycle (Birkeland et al., 2010). Enolase has also been suggested to regulate host-pathogen interactions through activation of the plasminogen system (Ayón-Núñez et al., 2018). Plasminogen is a proenzyme of the fibrinolytic system, in which plasmin is produced (Vassalli et al., 1991; Castellino and Ploplis, 2005). Once activated, plasmin cleaves extracellular matrix components, such as fibrin, fibronectin, laminin, collagen, elastin and proteoglycans, among others (Smith and Marshall, 2010). Host-derived plasmin can increase the pathogenicity of several protozoan parasites other than Giardia (Ayón-Núñez et al., 2018).

Although current evidence shows that Gd-eno plays a role in the encystation of trophozoites (Castillo-Romero et al., 2012) this enzyme might be involved in the regulation of carbohydrate metabolism. As multiple isoforms of enolase are known to be expressed in other parasitic protozoa (Bolten et al., 2008), it is possible that Gd-eno might have moonlighting functions associated with its single copy gene not being developmentally restricted.

As Gd-eno is expressed by Giardia and is released into culture medium upon interaction of trophozoites with epithelial cells (Ringqvist et al., 2008) we hypothesize that, under particular conditions, this enzyme can act as an activator of plasminogen in host epithelial cells and induce the degradation of tissue extracellular matrix via the serine protease plasmin, as seen in bacteria and tissues (Peetermans et al., 2014). In the present study, we tested this hypothesis and explored whether enolase contributes to Giardia’s virulence.

## Materials and methods

### Parasite culture

For all experiments, G. duodenalis trophozoites (WB strain, assemblage A, ATCC # 30957) were grown at 37°C in 15 mL conical bottom tubes in TYI-S-33 modified medium (Keister, 1983) containing 10% v/v heat-inactivated bovine serum (HyClone) with 1% antibiotic/antimycotic mixture (HyClone). Trophozoites were harvested at logarithmic growth phase by cooling the tubes in an ice-water bath for 1h and detached trophozoites were collected by centrifugation at 750 x g for 10 min at 4°C; the cell pellet was washed 3 times with phosphate-buffered saline (PBS; pH 7.4), and number of trophozoites determined using a Neubauer chamber (haemocytometer). The trophozoite concentration was adjusted according to the assays performed.

### Gene cloning and protein expression of rGd-eno

The open reading frame of the G. duodenalis gene encoding enolase (Gd-eno) (gi|237688745) was amplified by PCR from genomic DNA from strain WB using specific primers: Eno_100_upper sense (5’-CAC CAT GGA GGC TCC GTC TAC G-3’) and Eno_100_lower antisense (5’-TCA CTT CCA GGC CTC GAA ACC A-3’). The pET100-ENO was sequenced to verify that the linked gene was the correct one by using Big Dye Terminator v3.1 Cycle Sequencing Kit (Thermo Fisher Scientific) and PCR product cloned into the bacterial plasmid pET100/D-TOPO using the directional cloning system (Invitrogen). The resultant plasmid construct was used to transform Escherichia coli TOP10 and BL21. The BL21 bacteria were grown in Luria-Bertani (LB) medium at 37°C to an absorbance value (A600nm) of 1; the expression of the recombinant protein (rGd-eno) was induced by addition of isopropyl-thio-D-galactosidase (IPTG) to a final concentration of 1 mM, and incubation was continued for 3 h, at which time the cells were harvested by centrifugation at 22,000 x g for 20 min. The recombinant protein carrying the His-tag was purified by metal-affinity chromatography using Ni-NTA agarose according to manufacturer’s recommendations (Invitrogen). The recombinant protein was then passed through the high capacity endotoxin removal resin (Pierce™, Thermo Fisher) to eliminate any possible bacterial enterotoxins.

To assess whether there was residual endotoxin activity in the purified rGd-eno, pellets from bacteria transformed with the plasmid pET 100/TOPO with the enolase insert were resuspended in Lysis Buffer (50 mM Na2HPO4, 300 mM NaCl, 0.25 Triton X-100; Protease Inhibitors Complete EDTA Free (Roche™) and 1 mM PMSF) and sonicated. The NiTA agarose column (QIAGEN) was prepared by washing with 10 ml of Lysis Buffer and the sample was passed through the column twice. The first washing of the column was carried out with lysis buffer and 0.25% Triton X 100. Two subsequent washings were performed with lysis buffer containing 30 mM and 50 mM of Imidazole, respectively. Finally, elution was performed using lysis buffer but with 200 mM imidazole in the presence of Protease Inhibitors Complete EDTA-free (Roche™). SDS-PAGE (10% gel) was used to resolve protein fractions from bacterial extracts. The selected fractions were subjected to dialysis using a 12-14 kDa cut-off membrane (SpectraPor) and immersed in 2 L of PBS for 24 h and then again for the same time. Finally, the collected fractions were passed through a column of the High-Capacity Endotoxin Removal Resin Kit (Pierce, from Thermo Fisher Scientific).

### Enolase enzymatic activity assay

rGd-eno activity was determined by measuring the oxidation of NADH using a coupled assay with pyruvate kinase and lactate dehydrogenase as previously described (Saavedra et al., 2005). Briefly, the enzymatic assay was performed at 37 °C containing assay buffer (50 mM imidazole, 10 mM Tris, 10 mM acetate, 10 mM MES at pH 7.0), containing 5 mM MgCl2, 1 mM ADP, 0.15 mM NADH, 1 mM 2PG, 10 Units pyruvate kinase, 10 Units lactate dehydrogenase and 1.64 μg of rGd-eno. The decrease in NADH absorbance was monitored at 340 nm using a diode-array Agilent 8451 spectrophotometer (Agilent Technologies). In all determinations of enzyme activity, a linear relationship between amount of protein and activity was measured. rGd-eno activity was recorded as μmole/min x mg of protein.

### Anti-rGd-eno antibodies production

Polyclonal antibodies against rGd-eno were produced in mice. Female BALB/c mice (n = 10; females; 9 weeks old) were each inoculated intraperitoneally, four times at weekly intervals with 6 µg of purified recombinant protein extracted from polyacrylamide gels. The animals used in this study were fed ad libitum with rodent pellets (Purina™) and purified water and kept under constant standard temperature, humidity, and using filtered air. The handling of mice was performed according to Mexican regulations (NOM-062-ZOO-1999) for the production, care and use of laboratory animals (UPEAL-CINVESTAV). Pre-immune sera were obtained from individual mice and then pooled prior to immunization. One week after the fourth immunization, immune sera were collected from individual mice and pooled. The titers and specificity of the antibodies were determined by ELISA assays and immunoblotting analyses against the 6xHis-Tag.

### Preparation of parasite total extracts

Trophozoites or encysting stages of G. duodenalis were cultured in 15 ml Falcon conical bottom tubes using TYI-S33 medium supplemented with 10% heat-inactivated bovine calf serum (HyClone) and 100 units/mL of penicillin, 100 µg/mL streptomycin sulfate and ?25 µg/mL amphotericin B. Trophozoites (~50 million trophozoites/ml) were detached on ice for 30 min washed two times with PBS (at 10°C) and resuspended in 20 mM Tris pH 8.3 with protease inhibitors (Mini Complete, Roche) then sonicated on ice for 4 cycles of 15 sec at 30 sec intervals. After this, 40 µl/ml of Triton X-100 (Sigma) at 10% in Tris 20 mM, pH 8.3, were added and the lysate incubated on ice for 15 min, sonicated again and centrifuged at 10,000 xg for 30 min at 10°C. Finally, the supernatant was collected and protein concentration was determined using a kit (BCA Protein Assay, Pierce, Thermo Fisher Scientific); samples of 30 µg of protein were subjected to Western blot analysis.

### Determination of the reactivity of the anti-rGd-eno antibodies by Western blot and localization of enolase in trophozoites by indirect immunofluorescence assays

The reactivity and specificity of anti-rGd-eno antibodies were determined by Western blot analysis. In this, 1 µg of the purified rGd-eno, or 30 µg of the following samples: whole protein extract from G. duodenalis trophozoites (T), whole protein extract from G. duodenalis encysting trophozoites (C), were used. As negative control, a recombinant protein Rad-52 from G. duodenalis (rGd-Rad-52) was used. The samples were mixed with 6X Laemmli buffer and then were subjected to electrophoresis in a 12% acrylamide gel (Laemmli, 1970). The separated proteins were transferred to a nitrocellulose membrane; the membrane was blocked with 0.1%TBS-T with 10% nonfat dried milk for 1h at RT. Membranes were incubated initially for 1h at RT with anti-rGd-eno antibody at a 1:5000 dilution and then for 1h with goat anti-mouse IgG antibody coupled to peroxidase (GAMP) at 1:10,000 dilution. Bands of reactivity were revealed using chemiluminescence ECL kit (Amersham™ Biosciences) following the manufacturer’s instructions. Anti-histidine antibodies were used to detect rGd-eno.

Localization of enolase in trophozoites was determined by indirect immunofluorescence assays (IIF). In these, 4x106 trophozoites in TYI-S-33 medium were incubated in a 24 microtiter plate containing coverslips for 40 min. Afterwards trophozoites were permeabilized by incubating for 15 min at -20 °C in a 1:1 methanol-acetone solution, followed by blockage of nonspecific sites with PBS containing 10% bovine serum for 30 min at 37°C. Then an overnight incubation with primary anti-rGd-eno antibody (dilution 1:400 in PBS-BSA 1%) was carried out at 4°C followed by another for 45 min at 37°C with FITC-conjugated goat anti-mouse IgG (dilution 1:400). Nuclei were stained with Hoechst (dilution 1:1000) for 15 min at 37°C The anti-tubulin antibody (TAT-1, kindly provided by Dr. Keith Gull, University of Manchester, UK) was used to stain the ventral disk. Trophozoites were then fixed with 2% paraformaldehyde (PFA) and the samples were analyzed using a confocal laser microscope system (Leica Microsystems, Germany).

The amount of Gd-eno secreted by trophozoites was determined by Western blot of culture supernatants of IEC-6 cells exposed to Giardia trophozoites. For this, known quantities of rGd-eno and supernatants from IEC-6 cultures were collected (volume 400 µl) and concentrated (10X) by centrifugation using columns for protein concentration (Amicon® 4ml Ultra, Merck). 32 µl of the final volume were Western blotted using the polyclonal anti-rGd-eno antibodies and a densitometric analysis was carried out. The secreted amount of enolase in supernatants was calculated considering the final volume of concentrated supernatants and the values obtained from the curve.

### Native gel electrophoresis

The presence of secreted enolase monomer or the recombinant protein was determined in distinct samples by protein gel electrophoresis under non denaturing conditions. In these, samples containing ~1 µg of purified rGd-eno or concentrated culture supernatants (concentrated by centrifugation using 4 mL filters Amicon® Ultra, Merck) were mixed with a 5X solubilizing buffer (60% glycerol, 156 mM trizma-hydrochloride, 0.001% bromophenol blue). Proteins were analyzed by SDS-free electrophoresis in stacking 5% acrylamide gels and in separating 9% acrylamide gels for 4-5h at 80V and 4°C. The gels were stained with a solution containing 0.1% Coomassie blue- (40% methanol, 10% acetic acid) and distained with a similar solution lacking the dye.

### Inhibition of trophozoite attachment to IEC-6 cell monolayers by anti-rGd-eno in interaction assays

For interaction assays, 5x105 IEC-6 ATCC Cat. No. CRL-1592 cells were grown to confluence in 24 well plates (Nunc™ well surface: 2 cm2) in DMEM medium supplemented with 10% heat-inactivated fetal calf serum (HyClone™) and with 1% of a solution containing 10,000 units/mL of Penicillin, 10,000 µg/mL Streptomycin Sulfate, and 25 µg/mL Amphotericin B in a 0.85% Saline (Caisson). For the interaction assays culture medium was removed from cultures of IEC-6 cells and DMEM medium with no serum was added before exposing the cells to 2x106 trophozoites for 2h at 370C. Interaction was carried out in the presence or absence of anti-rGd-eno antibody at 1:250 dilution and 1:500 dilution. No adhered trophozoites in the media were collected by washing the wells with PBS at 37° C and processed with lysis buffer (SDS and HEPES), then the lysate was incubated with SYTOX Green and fluorescence emission at 523 nm of SYTOX Green-DNA bound was determined. To infer the amount of non-attached parasites, the same lysis procedure was carried out in order to determine the total emission of 2x106 trophozoites (100%) which was the number of parasites added per well.

### Plasminogen binding assay

To test the ability of enolase to bind human plasminogen, 1 and 5 µg of human plasminogen (Athens) were spotted onto nitrocellulose membrane. In addition, 1 µg of G. duodenalis 6His-Rad50 and 1 µg BSA were also spotted and used as negative controls. After the dots were dried, the membrane was blocked with 5% nonfat dried milk in PBS for 40 min at room temperature, washed 5 times with PBS and incubated with 10 µg rGd-eno for an additional hour. Binding of proteins was assessed by incubation with anti-rGd-eno antibody at 1:5,000 dilution in PBS followed by incubation with goat anti-mouse IgG coupled to peroxidase (GAMP) at 1: 10,000 dilution in PBS for 1h at RT. The membrane was washed 5 times with PBS and then developed by chemiluminescence ECL kit (Amersham™ Biosciences) according to manufacturer´s instructions.

### Plasmin activity assay

Determination of plasmin activity was done using the specific chromogenic plasmin substrate D-Val-L-Leu-L-Lys-p-nitroanilide (S-2551, Chromogenix). Briefly, a reaction in PBS containing 450 µM S-2551 substrate, 1 µg human plasminogen (Pl), 1 µg rGd-eno (reference glycolytic activity: 58 μmol/min x mg of protein) or 0.14 U streptokinase (Stk) were incubated for 15 min at 37°C in agitation (Thermomixer, Eppendorf). Each 5 min, samples of 50 µL were collected and the absorbance at 405 nm was determined; these values represent the activity of plasmin on the chromogenic substrate. To determine the direct effect of parasite’s enolase on the plasmin activity, 1x106 trophozoites were added to the reaction but in the absence of rGd-eno. In addition, enolase on the surface of trophozoites was blocked with anti-rGd-eno antibodies (1:150) prior to perform the interaction assays.

### Inhibition of plasmin activity by ϵ-aminocaproic acid

To evaluate the specific activation of plasmin by Gd-eno lysine residues, competition experiments were performed using the lysine analogue ϵ-aminocaproic acid (ϵ-ACA) (Sigma). Briefly, in vitro reactions to determine plasmin activity were carried out as described above, but in the presence of different concentrations (25 mM to 100 mM) of ϵ-ACA diluted in water. Reactions were incubated 15 min at 37°C and then, plasmin activity was determined by absorbance at 405 nm.

### Bioinformatics analyses

To identify domains and residues potentially involved in enolase catalysis (i.e. Mg2+ binding, substrate binding/protonation sites), interaction with plasminogen or variant residues amongst G. duodenalis assemblages, an alignment of the entire amino acid sequence of Gd-eno reported in GiardiaDB (http://giardiadb.org/giardiadb/; ORF GL50803_11118) was carried out with sequences from human, protozoan and yeast enolases using the CLUSTALW 2.1 software (http://www.genome.jp/tools/clustalw/) with default parameters. Further, the protein structure of Gd-eno was predicted by homology modeling using the Swiss Model server (https://swissmodel.expasy.org/) and three active conformations were obtained: “inactive” (without ion ligand, i-Gd-eno), “open” or “partially active” (with one Mg2+ atom, pa-Gd-eno) and “closed” or “fully active” (with two Mg2+ atoms, fa-Gd-eno) (Schreier and Höcker, 2010). This was based on the fact that recombinant or endogenous Gd-eno forms were experimentally used either purified or from trophozoite lysates, culture supernatants or interaction supernatants by monospecific antibodies forms, where the milieu varied in Mg2+ content. The stereochemical quality of all Gd-eno models were first evaluated by GMQE and QMEAN scores, then validated by the Ramachandran plot tool of the Discovery Studio 4.1 Client software to ensure its utility in further protein-protein docking analyses with crystal structures of human plasminogen type II (PDB ID: 4dur). These latter studies were carried out using the ClusPro platform (https://cluspro.bu.edu/) and the 15 most balanced complexes for each active conformation of Gd-eno were considered to select the optimal docking complex using as reference the most balanced complex of crystallized human enolase 1 (PDB ID: 3ucc) with the homologous plasminogen type II crystal structure indicated above. Visualization and editing of all protein structures and complexes were carried out using the UCSF Chimera™ v.1.10.1 software.

### Effect of rGd-eno on IEC-6 cell monolayers

IEC-6 cell monolayers were grown to confluence at 37° C in a 24 well plate using DMEM medium supplemented with 10% heat-inactivated fetal calf serum (HyClone™) and 1% of antibiotic-antimycotic solution containing 10,000 units/mL of Penicillin, 10,000µg/mL Streptomycin Sulfate, and 25µg/mL Amphotericin B in a 0.85% Saline (Caisson). Cell monolayers were incubated for 30, 60, 90 and 120 min with 100 μg/mL of rGd-eno. Activity of the purified enzyme was 58 μmol/min x mg of protein. After this time, cultures were washed and fixed according to the analysis to be performed. For Nomarski optics analysis, cells were fixed with PFA and observed using an Axioskop 40 Zeiss microscope and micrographs were analyzed with AxioVision Rel 4.8 software. For scanning electron microscopy, glutaraldehyde-fixed IEC-6 monolayers samples were dehydrated with increasing concentrations of ethanol, critical-point dried using a Samdri 780 apparatus (Tousimis, Rockville, Maryland, USA), coated with gold with a JEOL JFC-1100 ion-sputtering device, and examined with a JEOL JSM-7100F scanning electron microscope (Tokyo, Japan).

To further analyze the effect of rGd-eno in cell monolayers, cell confluence experiments were carried out as follows. Cells monolayers were grown in a 6 wells plate in 3 mL supplemented DMEM medium at 37° C5% CO2 to a final confluence of 50%, 70% and 100%. Then 100 μg of rGd-eno (reference glycolytic activity 58 μmol/min/mg of protein) were added to each well and after 180 min of interaction, cells were fixed and analyzed by microscopy. To analyze the inhibition of rGd-eno activity, different concentrations of anti-rGd-eno antibody (1:50, 1:100, 1:500 and 1:1000) diluted in DMEM media were added to the interaction assays of rGd-eno with IEC-6 epithelial cells. After 3h of incubation, cells were fixed with 2% PFA and then observed using an Axioskop 40 Zeiss microscope and micrographs were analyzed with AxioVision Release 4.8 software. The results were compared with the ones observed in control interactions assay in which no antibody was added.

### Biochemical determination of Caspase 3 activity

Caspase 3 activity was determined using a Caspase 3 assay buffer that contained: 20 mM Hepes, 5 mM DTT, 2 mM EDTA and 0.1% Triton X-100 at pH 7.4. For each determination, the buffer assay was supplemented with 50 μL of the cell lysate and 1 μ M Ac-DEVD-AMC (caspase-3 substrate) with or without 2 μM Ac-DEVD-CHO (caspase-3 inhibitor). The reactions mixtures were incubated at room temperature for 90 min. Caspase-3 activity was calculated by subtracting the fluorescence signal obtained when the substrate and inhibitor were mixed and compared with the reaction mixture that contained only the substrate. The corrected signal obtained from cells incubated with Staurosporine (STS) was considered as 100% response for caspase production (Benítez-Rangel et al., 2011; Benítez-Rangel et al., 2015).

### Annexin V binding assays

IEC-6 cells were grown on coverslips in DMEM at 37°C and 5% CO2 and when confluence was reached, the monolayers were exposed to purified rGd-eno at a final concentration of 100 μg/mL (reference glycolytic activity 58 μmol/min xmg of protein) for 2 h. Negative controls were IEC-6 cells incubated with DMEM alone and positive controls were IEC-6 cell monolayers exposed to 50 µM H2O2, cells without enolase were then incubated for 2h under the same conditions. After this, coverslips were treated with Annexin V-FITC conjugate (BioVision, Milpitas, Ca, USA) for 5 min following the manufacturer´s instructions, fixed with 2% PFA for 1 h, washed once with PBS and analyzed using an Axioscope Zeiss Zeiss epifluorescence microscope. For quantification by flow cytometry, cells were collected and exposed to the treatments mentioned above an analyzed with the Annexin V-FITC Apoptosis Kit (BioVision, Milpitas, Ca, USA) following the manufacturer’s instructions.

### Immunohistochemistry assays

Immunohistochemistry assays were performed for Caspase 3, Caspase 8, BID (BH3-interacting domain death agonist) and AIF (Apoptosis-inducing factor) proteins following standard procedures. Confluent IEC-6 monolayers were treated with rGd-eno at the concentration and times previously described. Cells were washed once with PBS and treated by 20 min with 4% PFA at room temperature to immobilize antigens while retaining cellular and subcellular structure. Cells were rehydrated following treatment with xylene, ethanol-xylene, ethanol 100%, ethanol 90%, ethanol 70% and water. Antigen retrieval was carried out at 90°C, in a bath, by treating the cells with sodium citrate (pH 6, 0.01M) for 20 min. Intrinsic peroxidase activity was eliminated by incubation of the cells with a 3% H2O2 -methanol solution at room temperature twice for 15 min. Blocking was performed using 2% normal porcine serum in PBS, for 1h in a wet camera at RT. The cells were then incubated with biotin for 15 min at room temperature and washed 3 times with PBS prior to the addition of the primary antibodies against the different proteins evaluated [anti-Caspase 3: (Cell signaling Technology, Rabbit mAb); anti-Caspase 8 (Cell signaling Technology, Rabbit mAb); anti-BID (Abcam, Rabbit mAb); anti-AIF (Cell signaling Technology, Rabbit polyclonal)], which were left overnight at room temperature. After 3 washes with PBS, universal biotinylated link (Dako, Carpinteria, CA) was added to the cells, and then incubated for 30 min with HRP-Conjugated Streptavidin and revealed with substrate diaminobenzidine (DAB). After analysis, cells were dehydrated and preserved.

### Detection of AIF and TNFα in IEC-6 cells or supernatants after interaction of with rGd-eno

Detection of AIF and TNFα was carried out by Western blot assays using antibodies against AIF and TNFα as follows. For AIF, Western blot analysis, after treatment with rGd-eno, IEC-6 epithelial cells were lysed with RIPA buffer and, equal amounts of proteins were separated by SDS-PAGE in a 10% polyacrylamide gel and transferred to a nitrocellulose membrane. Then, membranes were blocked and incubated overnight with anti-AIF at 1:1500 dilution (Cell Signaling) in 1.2% milk-PBS solution. Membranes were incubated with goat anti-mouse IgG coupled to peroxidase diluted 1:10,000 in PBS for 1h at RT. Detection was carried out using a chemiluminiscence kit. For detection of TNFα, 50 µL of supernatants of interaction were diluted 1:5 in 5X Laemmli buffer with 1% β-mercaptoethanol and boiled for 5 min. Samples were then separated by SDS-PAGE on 10% polyacrylamide gel and blotted to PVDF membranes. Membranes were incubated with blocking buffer (3% v/v casein in 10 mM Tris-HCl pH 8.0, 150 mM NaCl, 0.05% v/v Tween-20), and then incubated overnight at 4°C with the primary antibody against TNF-α (1:1000, Santa Cruz Biotechnology) diluted in blocking buffer. Thereafter, membranes were incubated for 1h with HRP conjugated donkey anti-goat IgG (Santa Cruz Biotechnology) antibody diluted at 1:10,000; after washing, reactivity was developed using the ECL™ prime Western blotting detection reagent (Amersham™, GE Healthcare, Buckinghamshire, UK). Chemiluminescence was detected in an EC3 Imaging System (UVP Bio lmaging Systems, Cambridge, UK). Protein band density was quantified by transmittance densitometry (UVP Bio Imaging Systems software, Cambridge, UK).

In other sets of experiments, the specific TNFα inhibitor TAPI-0 (TAPI-0, CAS: 143457-40-3, Santa Cruz Biotechnology) was used. Briefly, interaction experiments of IEC-6 monolayers with 60 μg/mL of rGd-eno (relative glycolytic activity 153 μmol/min x mg of protein) in DMEM were developed as previously mentioned. Then TAPI-0 was added to the interaction assays to a final concentration of 100 mM and the epithelial cell damage was analyzed by Nomarski optics as described above. Additional studies were carried out using the IκBα phosphorylation and NF-kB inhibitor BAY 11-7082 (Cayman, Chemical). Briefly, IEC-6 cells were cultured in a 2 cm2 coverslips in a 24 well plate and the interaction procedure with rGd-eno was carried in the absence or presence of the inhibitor at different concentrations (40 mM and 60 mM). After 3h of incubation, the cells were fixed with 2% PFA and then observed using an Axioskop 40 Zeiss microscope and micrographs were analyzed with AxioVision Rel 4.8 software.

### Statistical analyses

All statistical analyses were performed using the statistical package GraphPad Prism V.5. Data were analyzed by Student’s T-test with a 95% confidence interval for significance. Results shown are the representation of at least of 3 independent experiments.

## Results

### Sequence-based comparison of Gd-eno with homologs from other organisms

Sequences representing enolase homologs from H. sapiens, S. cerevisiae and T. brucei (accession numbers Homo sapiens AAB59554.1; S. cerevisiae: AAA88713; T. brucei: AAF73201.1) were compared with Gd-eno (accession number G duodenalis: EES98422). These enolases shared 51% identity over 96-98% of the amino acid sequence, with some insertion/deletion events (**Supplementary Figure S1**). In the Gd-eno, several amino acid residues were inferred to be involved in PEP binding (H170, E222, K361, H389 and K412) as well as Mg2+ binding (S44, D257, E306 and E336) (Castillo-Romero et al., 2012). Four additional amino acids implicated in PEP binding (i.e. A43, E179, R390 and S391) were identified based on structural predictions of clashes/contacts of Gd-eno with its substrate (**Supplementary Figure S1**). In addition, the plasminogen interacting domain (PID) of Gd-eno dimer (S261-N274) (Aguayo-Ortiz et al., 2017) and three Lys residues involved in primary interaction with human plasminogen (K361, K412 and K342) were recognized (**Supplementary Figure S1**). The sequence of Gd-eno also contains two residues that allow the formation of the anti-parallel homodimer, E25 and R429 (E20 and R414 in human α-enolase), in accordance with the human homolog. Regarding intra-species polymorphism of Gd-eno, the amino acid sequences representing five Giardia strains and three assemblages (A, B and E) displayed up to 31 variable residues (cf. legend of **Supplementary Figure S1**), 16 of which were displayed in assemblage B (with reference to assemblage A).

### Cloning and expression of rGd-eno

The nucleotide sequence (1338-bp) cloned into the pET100/D-TOPO was identical to a reference sequence from G. duodenalis Genbank accession no. EES98422 (**Supplementary Figure S2**). The purified recombinant protein (rGd-eno) was enzymatically functional, with a specific activity of 58 μmol/min x mg of protein – similar to that reported for recombinant enolase of Entamoeba histolytica (Saavedra et al., 2005).

### Specificity of anti-rGd-eno antibodies and localization of enolase in Giardia trophozoites and quantification of secreted enolase

**Figure 1:**
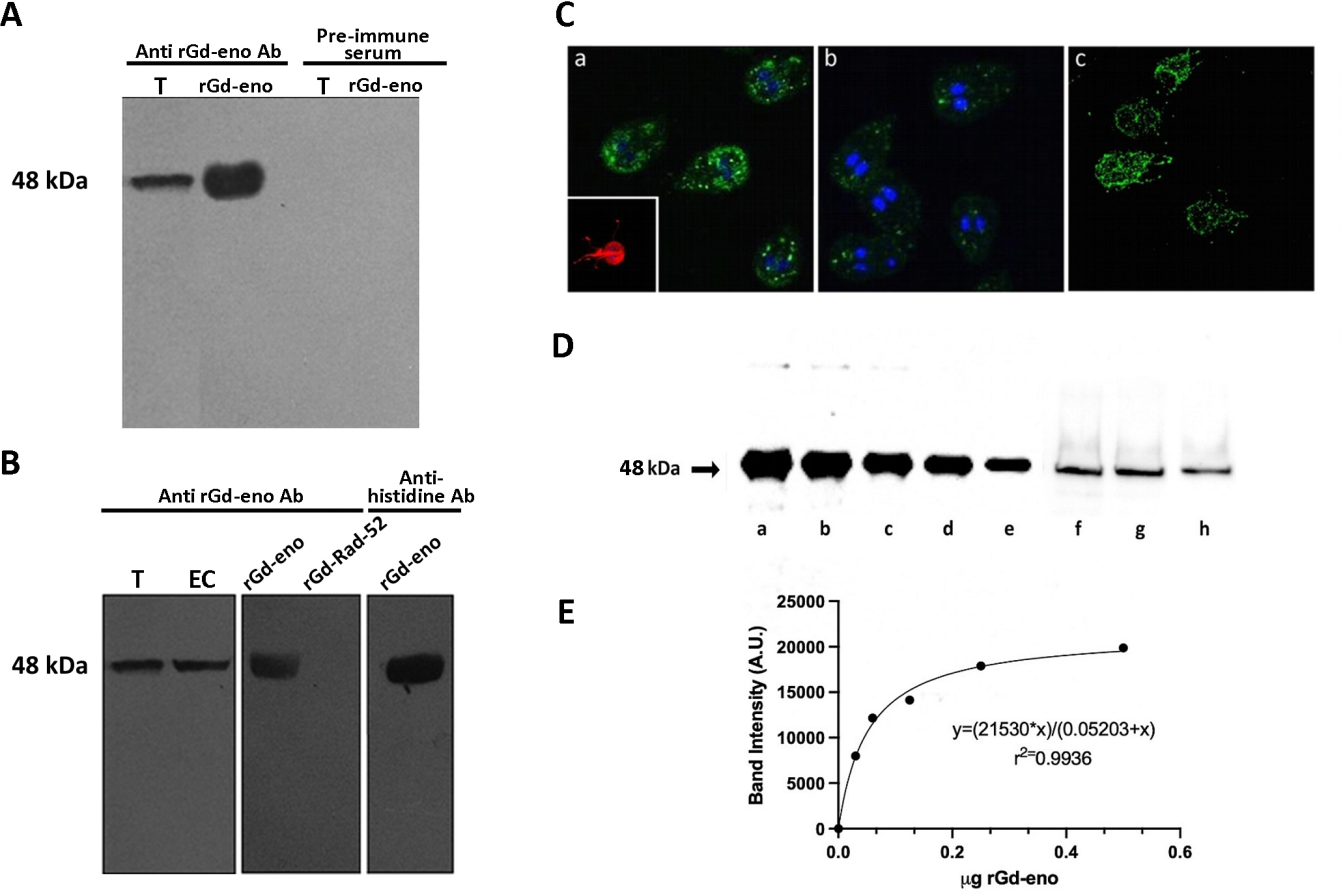
Specificity of anti-rGd-eno antibodies, subcellular localization of *Giardia* enolase and determination of secreted enolase in trophozoite interaction with IEC-6 cells. **(A)** Reactivity of anti-rGd-eno and pre-immune serum samples. Total extracts from *G duodenalis* trophozoites (T) and purified rGd-eno **(b)** were separated by SDS-PAGE, and the membranes probed with anti-rGd-eno and pre-immune serum. **(B)** Total extracts from trophozoite (T) and encysting cells (EC), rGd-eno and a non-related recombinant protein from *Giardia* named rGd-Rad52 were probed with anti-rGd-eno antibodies. Western blotting the membranes with anti-histidine antibodies directed against the his-tag present in rGd-eno was used as positive control. **(C)** Indirect immunofluorescence for endogenous Gd-eno was performed in trophozoites. Gd-eno is enriched at the ventral disk region and in small vesicles in the cytoplasm (a and b); (insert: staining of the adhesion disk with anti-tubulin antibody). Cytosolic detection was performed in permeabilized parasites and surface detection was carried out in fixed non-permeabilized trophozoites. Gd-eno (c) is localized in the entire parasite surface and flagella in fixed trophozoites. **(D)** Quantification of secreted Gd-eno. Concentrated supernatants of IEC-6 cells interacted with trophozoites for 1h **(f)** 2h **(g)** and 3h **(h)** and different quantities of rGd-eno: 500 ng **(a)**, 250 ng **(b)**, 120 ng **(c)** 60 ng **(d)** and 30 ng **(e)** were Western blotted with anti-rGd-eno antibodies. **(E)** The regression standard curve shown in D was generated from the densitometric analysis carried out in the protein bands and used to determine the concentration of secreted enolase present in the supernatants. Results are representative of two independent experiments.

The specificity of polyclonal antibodies generated against rGd-Eno was tested by Western blotting total extracts obtained from trophozoites and encysting trophozoites, the recombinant protein rGd-eno and a non-related recombinant protein referred as rGd-rad52 from G. duodenalis that was used as negative control (**Figure 1A**). As observed in **Figure 1A** a single band with molecular weight of 48 kDa was recognized by anti-rGdeno in total extracts obtained from trophozoites as well as with the purified rGdeno while no reactivity was observed either with total trophozoite extracts or with rGd-eno using the pre-immune serum (**Figure 1A**). The 48 kDa protein was detected also in encysting cells (**Figure 1B**) but no reactivity was observed with the rG-rad52 protein. As expected, commercial anti-histidine antibodies reacted with the 48 kDa band corresponding to the rGd-eno that is fused to the his-tag (**Figure 1B**). Thus, our results show that the antibodies obtained against rGd-eno specifically recognized both the native and recombinant protein. Next these antibodies were used to localize Gd-eno in permeabilized Giardia by indirect immunofluorescence (**Figure 1C**). Gd-eno was localized enriched at the region of the ventral disk and within small, rounded and well defined vesicles in the cytoplasm of trophozoites **(Figure 1C-b**). In non-permeabilized trophozoites, enolase was distributed on the whole parasite surface, including the flagella (**Figure 1C-c**). In conjunction, our results demonstrate that Gd-eno is localized to intracellular and epicellular components of trophozoites.

Specific experimental conditions can affect biological processes, including protein secretion. Therefore, our next goal was to evaluate the amount of Gd-eno secreted by trophozoites when interacting with IEC-6 cells. To such purpose, concentrated culture supernatants (concentrated by centrifugation using 4 mL filters Amicon® Ultra, Merck) were Western blotted using the anti-rGd-eno antibodies (**Figures 1Df–h**). A linear regression analysis of the densitometric values obtained from defined quantities of rGd-eno (**Figures 1Da–e**) were used to determine the concentration of the Gd-eno released. A calibration curve was obtained which displayed a hyperbolic trend and a good correlation coefficient (R2 > 0.99), allowing to estimate by extrapolation the quantities of Gd-eno secreted in concentrated supernatants. As shown in **Figure 1E**, it was estimated that approximately 25 ng (**Figure 1 Df**), 21 ng (**Figure 1Dg**)) and 14 ng of Gd-eno (**Figure 1Dh**) were secreted during the throphozoite-IEC-6 interaction respectively. Thus, considering that 32 µl of concentrated supernatant were Western blotted, the final values of secreted enolase were calculated for the final volume (40 µl) of concentrated supernatants. Consequently, our approximate values were as follows: 1h-31 ng; 2h-26 ng and 3h-17ng. The values calculated in our experimental conditions corresponded to 2 x 10 6 trophozoites added to the IEC-6 cells and the specific activity of rGd-eno determined in our experiments was used as reference for further experiments where rGd-eno was incubated with epithelial cells.

### Gd-eno is secreted as a monomer by G. duodenalis trophozoites

**Figure 2:**
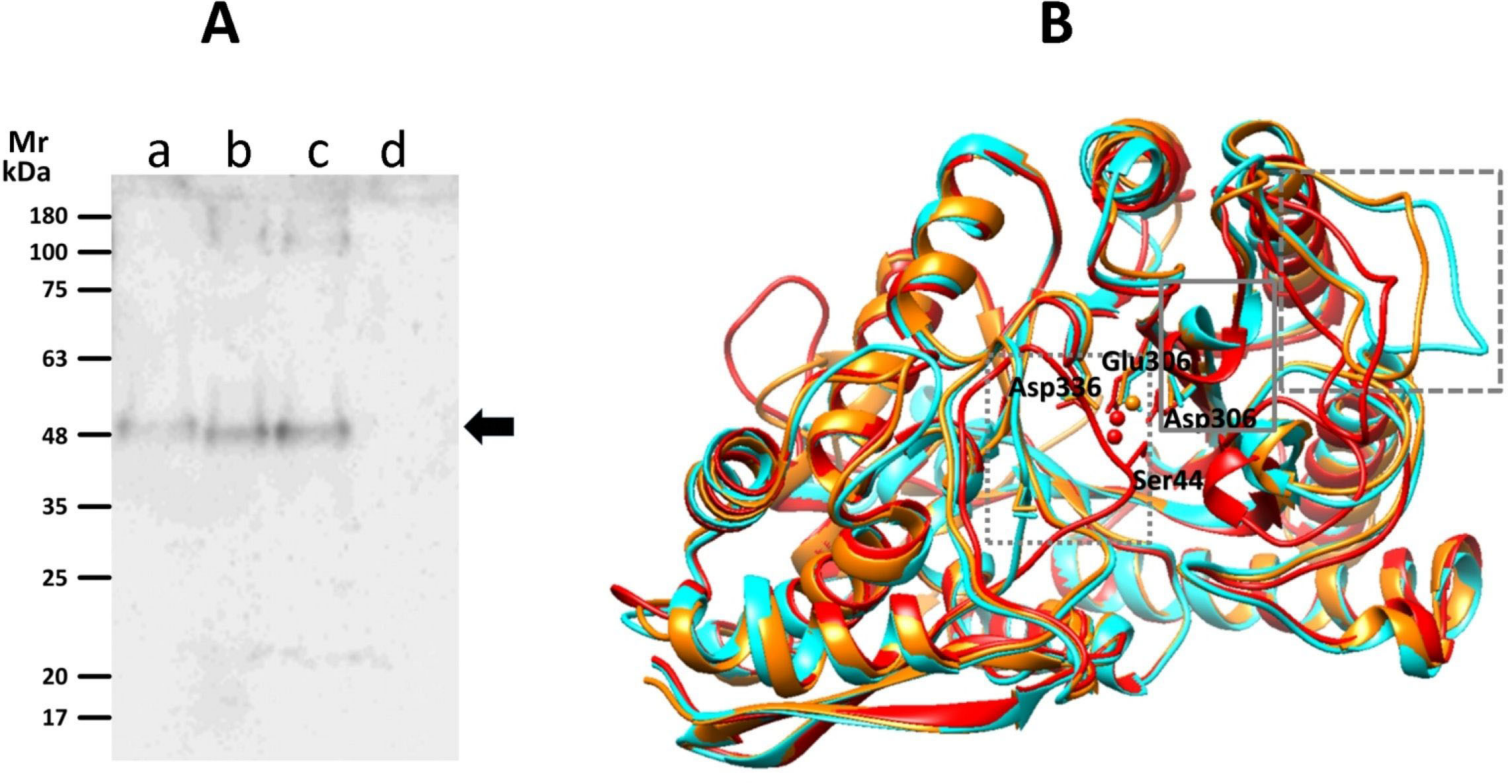
Detection of Gd-eno by native gel electrophoresis and active conformations of its monomer. **(A)** Western blot analysis using polyclonal anti-rGd-eno antibodies. Protein samples are as follows; **(a)** supernatants collected from a 2h trophozoite culture; **(b)** affinity chromatography purified rGdEno (1 µg); **(c)** supernatants collected from a 2h trophozoite-IEC6 co-culture, 15X-concentrated; **(d)** supernatant collected from a 2h IEC6 culture 10X-concentrated. Molecular weight markers (m) are at the left of the figure. **(B)** Protein modeling of Gd-eno (GL50803_11118) in “inactive” (none Mg^2+^-bound, displayed in cyan), “partially active” (one Mg^2+^ bound, displayed in orange) and “fully active” (two Mg^2+^ bound, displayed in red) conformations. The squares with doted, continuous and dashed gray lines mark the closing of loop 1, loop 2 and loop 3 domains, respectively. Mg^2+^ ions are depicted in *ball* conformation and amino acid residues involved in Mg^2+^binding are indicated and shown in *ball-and-stick* conformation.

Enolase exhibits different homocomplex orders, such as monomers, dimers and octamers (tetramers of dimers) (Lu et al., 2012; Wu et al., 2015), and in our results we detected an endogenous Gd-eno of 48 kDa in trophozoites. Therefore, we determined the aggregation states of the enzyme present in culture supernatants. Under non-denaturing conditions, antibodies recognized a band of ~48 kDa in supernatants collected from trophozoites cultured alone or co-cultured with IEC-6 cells that corresponded to the monomeric form of Gd-eno (**Figure 2Aa–c**). The secretion of the monomeric form of Gd-eno was higher in the presence of IEC6. No bands were observed in supernatants from IEC-6C cell monolayers (**Figure 2Ad**).

Since Gd-eno was secreted as a monomer, the active conformation of this molecule was evaluated by bioinformatic analyses. Models were predicted using distinct optimal templates: (a) α-enolase of Toxoplasma gondii (PDB ID: 3otr) for i-Gd-eno that showed scores QMEAN = -1.46 and GMQE = 0.77, (b) Saccharomyces cerevisiae enolase (PDB ID:1ebh) for pa-Gd-eno with scores of QMEAN = -0.82 and GMQE = 0.76, and (c) Homo sapiens neural enolase (PDB ID: 3ucc) for fa-Gd-eno with scores QMEAN = -0.44 and GMQE = 0.77 (**Figure 2B**). In the latter model, similar results were obtained using H. sapiens enolase 1 (PDB ID: 2psn) as template. In all models, Ramachandran plots predicted that >99% of residues were within allowed zones in ψ-ϕ angle quadrants, indicating a high stereochemical quality of the models. As shown by structural alignment of all three models (**Figure 2B**), the expected closing of connecting loops L1, L2 and L3 were well defined. These conformations were considered as representative of extracellular/secreted Gd-eno and rGd-eno in further molecular docking studies.

### Gd-eno participates in trophozoite attachment to epithelial cells

**Figure 3:**
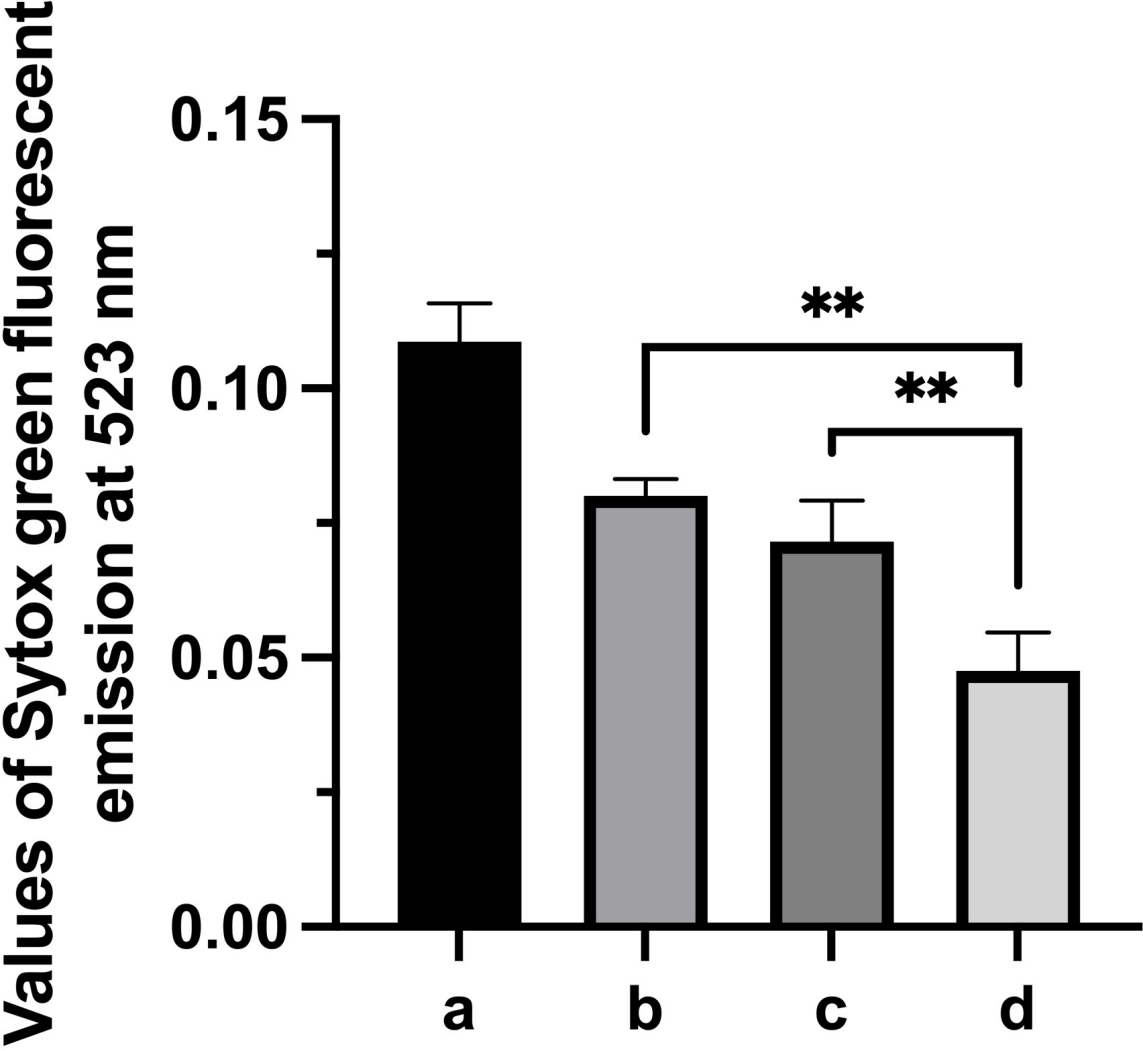
Effect of anti-Gd-eno antibodies in the attachment of *Giardia duodenalis* trophozoites to IEC-6 monolayers. IEC-6 epithelial cells monolayers were co-cultured with 2 x 10^6^ trophozoites that were pre-incubated with anti-rGd-eno at **(B)** 1:250 dilution, **(C)** 1:500 dilution. **(D)** Control co-cultures lacking antibody. Attachment of trophozoites was determined by Sytox Green fluorescent emission at 523 nm considering as reference the values obtained of fluorescence emission generated by 2x10^6^ cutured trophozoites **(A)**. Data shown is the mean average of three independent experiments performed by quadruplicate. *** P<0.05 in relation to control cultures lacking antibody and between antibody dilutions. **P<0.05.

Based on the observations that Gd-eno was abundantly expressed in the parasite`s surface and enriched in the region of the trophozoites´ventral disk the role of Gd-eno in the interaction with IEC-6 epithelial cells was analyzed. To this end, surface expressed Gd-eno on trophozoites was blocked by incubating the parasites with anti-rGd-eno antibody previous to the interaction with IEC-6 epithelial cells. Then the value of the fluorescence emission of SYTOX green-DNA complex from untreated, cultured trophozoites (2x106) which was 0.10873 ± 0.0071 (100%) was taken as reference (**Figure 3A**). In this manner, treatment of trophozoites with anti-rGd-eno antibody at 1:250 and 1:500 dilutions resulted in a significantly higher emission of SYTOX Green bound DNA values of 0.08003 ± 0.0032 and0.07162 ± 0.0076. [**Figure 3B** (74%) and 3c (66%] respectively], when compared with the control cultures in which no antibody was used [**Figure 3D** value of 0.04761 ± 0.0071 (44%)] representing a lower attachment ability of parasites to cell monolayers. These results suggest that Gd-eno participates in trophozoite attachment to epithelial cells.

### rGd-eno specifically binds plasminogen and enhances plasmin activity

**Figure 4:**
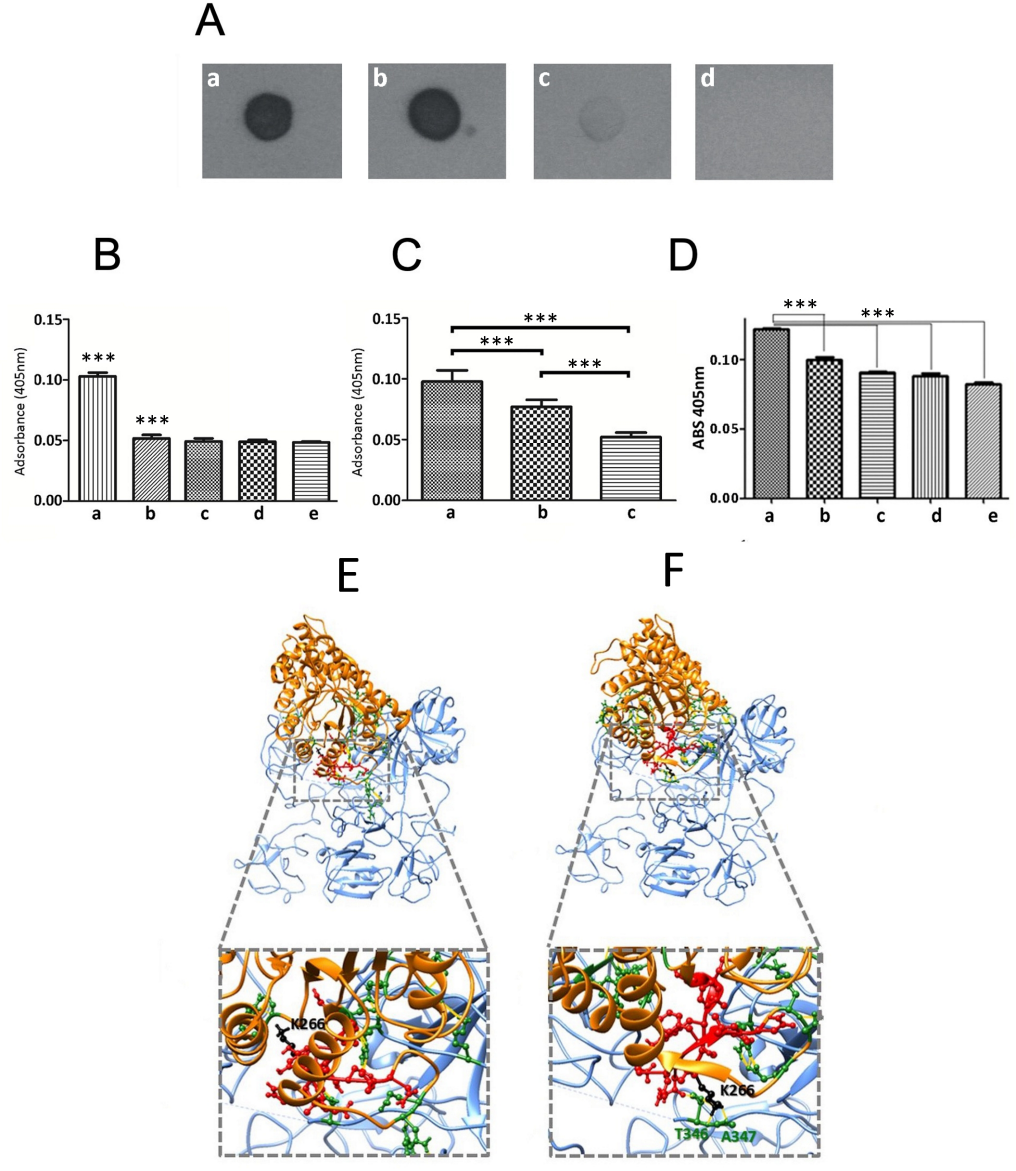
rGd-eno binding to plasminogen and activation of plasmin by rGd-eno and trophozoites. **(A)** Binding of rGd-eno to plasminogen was determined by dot blot assays. 1 µg **(a)** or 5 µg **(b)** of human plasminogen (PI) were added to the nitrocellulose membrane. 1 μg of 6His-GdRad50 **(c)** and 1 µg bovine serum albumin (BSA) **(d)** were used as controls. Membranes were incubated with 10 µg of rGd-eno in PBS, washed 3 times with PBS/tween and then blotted with anti-rGd-eno (1:5000). **(B)** Quantification of the plasmin activity induced by rGd-eno. **(a)** *in vitro* reaction including Streptokinase (Stk), Pl and S-2551 and rGd-eno, **(b)** *In vitro* reaction including Stk, Pl and S-2551. Control samples included, **(c)** S-2551 in PBS, **(d)** Stk and S-2551 and, **(e)** rGd-eno, Pl and S-2551. The data shown is the average of three independent experiments at 15 min reaction, each performed by triplicate. **(C)** Differential activity of plasmin induced by trophozoites. **(a)** 1x10^6^ trophozoites incubated with the reaction mixture containing Stk, Pl, S-2551; **(b)** trophozoites treated with anti-rGd-eno antibody previous to the interaction assays added to the reaction mixture **(c)** no trophozoites added to the reaction mixture. The data shown is the average of three independent experiments, each performed by triplicate. **(D)** Effect of ϵ-ACA on plasmin activity induced by rGd-eno. Protease activity was determined in the presence of rGd-eno, Pl and increasing concentrations of the lysine analog ε-ACA. The use of the specific competitor ϵ-ACA significantly reduced the reactivity of S-2551 *in vitro* in a concentration dependent manner. No addition of ϵ-ACA **(a)**, 25mM **(b)**, 50mM **(c)**, 75mM **(d)** and **(e)** 100mM of ϵ-ACA were added in these assays. Asterisks (**) indicate the statistical significance of P<0.05 in relation to control. **(E, F)** The putative binding of the Gd-eno monomer to human plasminogen was analyzed by protein-protein docking using the crystal structure of HsPlsII (PDB ID: 3ucc; displayed in blue) as ligand and the protein structures of Gd-eno monomer in “partially active” **(E)** and “fully active” **(F)** conformation (displayed in orange). The PBD of Gd-eno (S261-N274) is displayed in *ball-and-stick* conformation and colored red. The central residue K266 is colored in black. Residues displayed in *ball-and-stick* conformation and colored green are involved in clashes/contacts (displayed as yellow lines) between the two proteins. ***P < 0.001.

To evaluate whether Gd-eno acts as a plasminogen receptor, we exposed increasing amounts of purified human plasminogen in a nitrocellulose membrane to rGd-eno. The results showed a clear interaction between rGd-eno and plasminogen (**Figures 4Aa, b**). No binding of rGd-eno with 6xHis-Rad50 was observed or BSA (**Figure 4Ad**). (**Figure 4 Ac**). Given that 6xHis-Rasd50 failed to interact with plasminogen, we concluded that the conditions used for the purification of rGd-eno, and the His-tag present in rGd-eno did not interfere with the association of both molecules (**Figure 4Ad**). Subsequently, we determined the transformation of plasminogen to its active form, the serine-protease plasmin. Thus, we evaluated the activity of plasmin on its chromogenic substrate S-2251 D-valyl-L-leucyl-lysine-p-nitroanilide (Chromogenix). The maximum time point of reaction was set at 15 min after incubation because at that time the reaction was saturated. As seen in **Figure 4B**, the activity of plasmin was enhanced in the presence of rGd-eno (**Figure 4Ba**) as compared with a samples lacking rGd-eno (**Figure 4Bb**). No plasmin activity was detected the control groups (Gd-rad50 and BSA; **Figures 4Bc, d**) These results demonstrated that Gd-eno is a human plasminogen receptor that enhances the protease activity of plasmin.

Subsequently, we carried out assays aimed to determine the presence of an extrinsic plasmin activator in trophozoites. For this purpose, trophozoites (2x106) were included in reactions containing the plasmin specific chromogenic substrate S-2551, human plasminogen and streptokinase (Stk) as an external activator of plasmin (**Figure 4C**). The treatment of trophozoites with sub-agglutinating concentrations of anti-enolase antibody (1:250) prior to reaction, showed a reduced plasmin activation (**Figure 4Cb**) when compared with non-treated trophozoites (**Figure 4 Ca**), or with the control reaction with no trophozoites or when trophozoites were treated with rGd-eno (**Figure 4Cc**). Controls, including trophozoites in the absence of Stk, did not show plasmin activity, suggesting that G. duodenalis trophozoites do not produce an extrinsic plasmin activator, as observed in other pathogens (Lähteenmäki et al., 2001). Further, the addition of 100 mM of the lysine analog ϵ-aminocaproic acid (ϵ-ACA) significantly inhibited the activation of plasmin, measured by the absorbance of the chromogenic substrate S-2551 in vitro (**Figure 4D**).

### Inferring the molecular interaction of Gd-eno monomer and HsPls

To obtain insights on the molecular interactions between Gd-eno and plasminogen, we undertook in silico structural docking analyses with crystal structure of dimeric human plasminogen II HSPIs (PDB ID: 4dur) z- the predominant type recruited to cell surfaces (Law et al., 2012). The most balanced Gd-eno-HsPlsII complexes from a set of 15 predictions (**Figures 4E, F**) revealed multiple clashes/contacts (<4 Å) with the target. In these models, Gd-eno monomer (ligand) in its “partially active” (**Figures 4E**) and “fully active” (**Figure 4F**) states display a similar ability to interact through its PBD (S261-N274) and K266 neighboring moieties with HsPlsII at the lysine-rich environment present at the serine protease (SP) and with Kringle (K3 and K4) domains of this protein. This interactive microenvironment is consistent with the inhibitory effect of the lysine analog, ϵ-ACA, on plasmin activity induced by rGd-eno (**Figure 4D**).

### rGd-eno induces damage to IEC-6 monolayers

**Figure 5:**
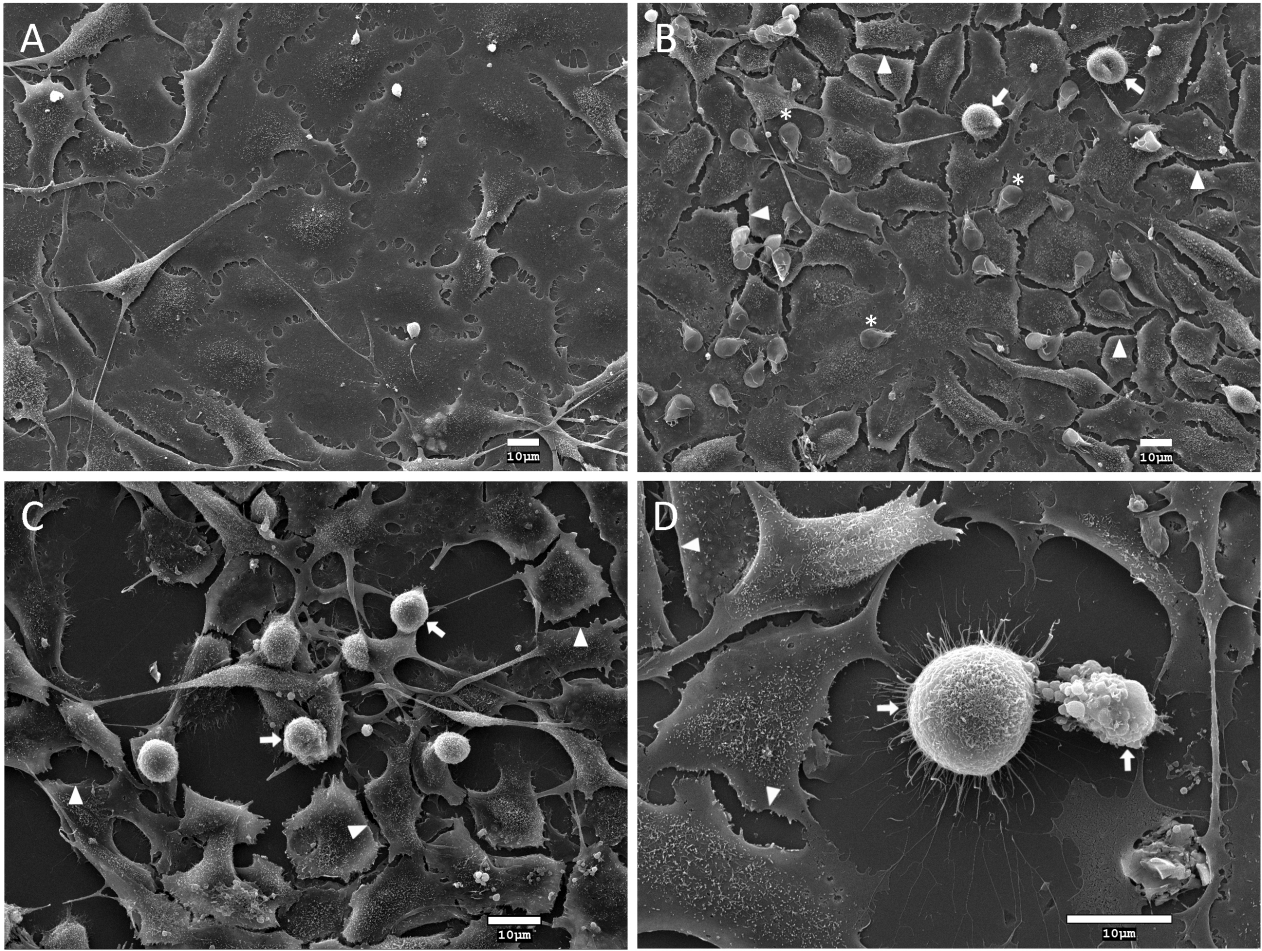
Analysis of the effect of *Giardia duodenalis* trophozoites and rGd-eno on IEC-6 epithelial cell monolayers. Intestinal epithelial cell monolayers of IEC-6 cells were incubated for 90 min with: **(A)** DMEM medium only; **(B)** *G duodenalis* trophozoites expressing surface Gd-eno; or **(C, D)** rGd-eno (100 μg/ml; relative glycolytic activity 58 μmol/minxmg of protein). Samples were processed for scanning electron microscopy. Representative micrographs are shown. IEC-6 monolayers exposed to trophozoites that express surface Gd-eno **(B)** or rGd-eno **(C, D)** displayed cell damage characterized by cell-cell separation (arrowheads), cell blebbing and cell shrinkage (arrows). Asterisks denote trophozoites. **(D)** A magnified view of cell blebbing and a shrinked IEC-6 cell.

Because rGd-eno activates the cysteine protease plasmin, we evaluated whether rGd-eno affected the integrity of IEC-6 monolayers. In IEC-6 cell monolayers exposed to rGd-eno, rounded cells with blebs that lost their cell-cell contact were consistently detected. In some areas, shrunken cells with vesiculated cytoplasm were observed (**Figures 5A–D**). This appearance was very similar to that described for epithelial cells undergoing apoptosis. Importantly, the treatment of the epithelial cells with an unrelated protein Rad-52 from G duodenalis, which was purified using a similar method as that used for Gd-eno, did not show any effect on cell morphology (data not shown). IEC-6 monolayers cultured in DMEM alone (**Supplementary Figure 3A**) or exposed to protein fractions purified from extracts from E. coli BL21 Star alone remained undamaged (**Supplementary Figure 3B**), while IEC-6 monolayers exposed to rGd-eno showed cell damage, as previously described. Thus, our results indicated that rGd-eno caused the alterations seen in IEC-6 cells (**Supplementary Figure 3C**). Interestingly, sparse monolayers were less susceptible to the damage induced by rGd-eno than confluent monolayers (see **Supplementary Figure 4**). These results suggested that Gd-eno has the potential to activate plasminogen, as has been reported for enolases of bacteria and tissues (Peetermans et al., 2014) However, further studies of epithelial cells are still needed to fully characterize the relationship between cellular plasminogen and its activation by Gd-eno.

### Anti rGd-eno antibody prevents the damage in IEC-6 cells that is mediated by rGd-Eno

**Figure 6:**
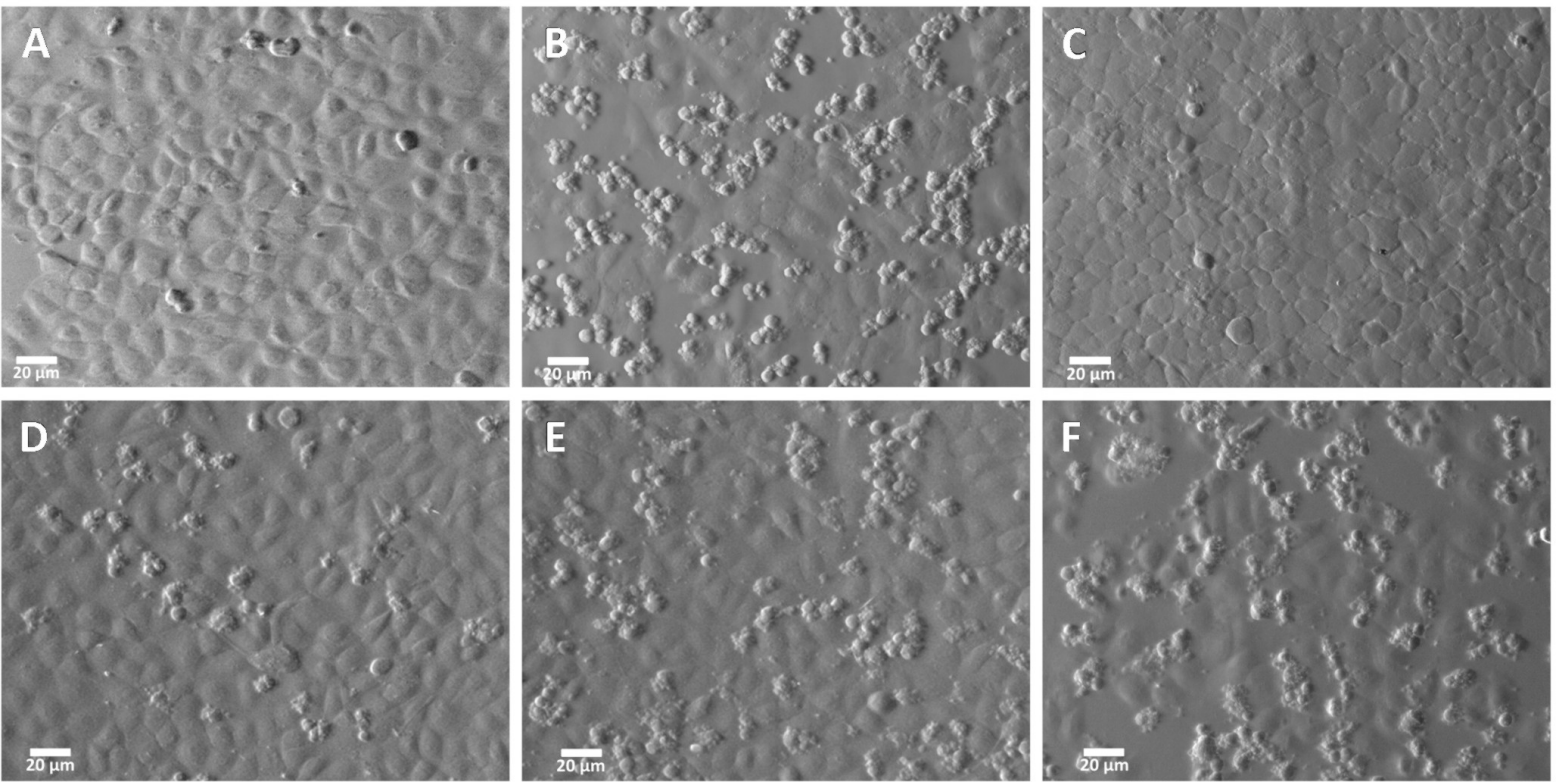
Inhibition of the damage induced by rGd-eno on IEC-6 epithelial cell monolayer by anti rGd-eno antibodies. IEC-6 cells were incubated with different antibody dilutions of anti-rGd-eno previous to the addition of rGd-eno (100 ug/ml enolase relative glycolytic activity 58 μmol/min x mg of protein). **(A)** negative control no enolase or antibody added; **(B)** addition of rGd-eno only; anti-rGd-eno added at **(C)** 1:50; **(D)** 1:250; **(E)**, 1:500 and **(F)** 1:1000 dilutions of anti rGd-eno antibodies. Images shown are representatives of three independent experiments.

To confirm the role of rGd-eno in the induction of cell death observed in IEC-6 cells, neutralizing experiments using anti- rGd-eno antibody were carried out. For this, increasing dilutions (1:50, 1:250, 1:500 and 1:1000) of anti-rGd-eno antibody were added to IEC-6 monolayers before the incubation with rGd-eno (100 μg/ml). As expected, no damage was detected in control monolayers (**Figure 6A**), while IEC-6 cells exposed to rGd-eno showed the cell-cell separation, blebbing and shrinkage (**Figure 6B**). In contrast, in epithelial cells previously incubated with anti-rGd-eno and then exposed to rGd-eno the cell damage was reduced in a concentration dependent manner (**Figures 6C–F**).

### Evidence that AIF and TNFα mediate cell death in IEC-6 exposed to rGd-eno

**Figure 7:**
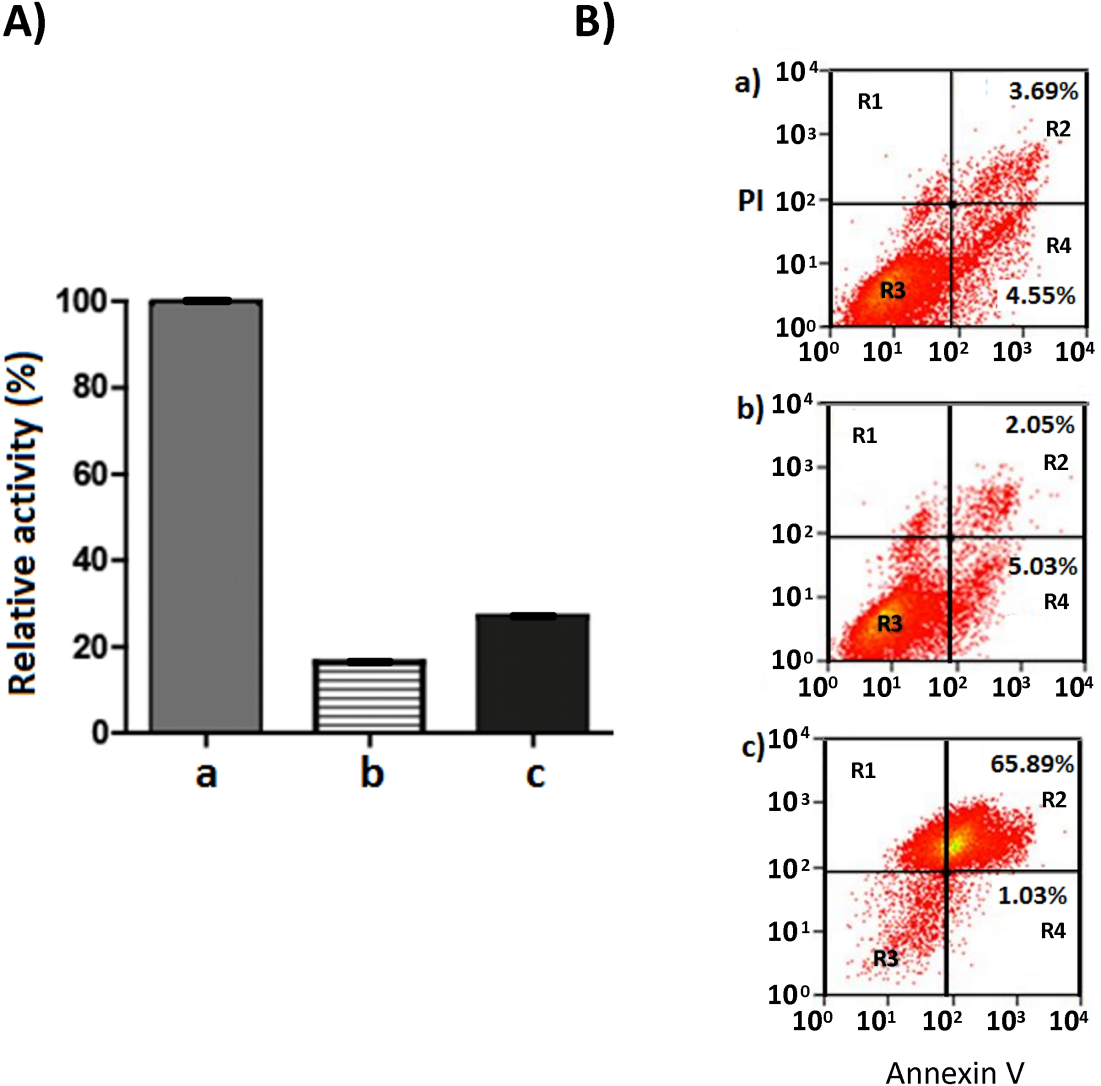
Caspase 3 activity in IEC-6 monolayers treated with rGd-eno and Annexin V-PI staining of IEC-6 cells exposed to rGd-eno. **(A)** Caspase 3 activity was determined in IEC-6 monolayers treated with rGd-eno (100 μg/ml; relative glycolytic activity 58 μmol/min x mg of protein) for 3h. Staurosporine-treated cells = positive control **(a)**. Control untreated cells **(b)**. IEC-6 cells treated with rGd-eno **(c)**. **(B)** Flow cytometry plots of Annexin V-PI staining of IEC-6 monolayers after 3h of exposure to **(a)** rGd-eno, **(b)** untreated control and c) H_2_O_2_-treated (control of apoptotic damage). R1: cells in necrotic damage (PI-positive), R2: cells in late apoptosis (Annexin V and PI-positive), R3: non stained cells, R4: cells in early apoptosis (Annexin V-positive). Percentages of cells in early and late apoptosis are indicated in each plot.

Next, we evaluated the mechanism triggered by rGd-eno to induce IEC-6 cell death in vitro. Initially, we explored the Programed Cell Death Type 1 by assessing caspase 3 activation in the epithelial cells (**Figure 7**). Despite of inducing cell death, rGd-eno treatment failed to activate caspase 3 in IEC-6 cells (**Figure 7c**). Further analyses of the activation of caspase 3 using immunofluorescence and immunohistochemistry assays did not reveal changes in the expression of the molecule in rGd-eno-treated cells versus non-treated cell monolayers (**Supplementary Figure 4**). To corroborate these findings, we assessed the presence of phosphatidylserine on the outer cell surface of epithelial cells. Flow cytometry readings determined that cells interacting with rGd-eno (100 µg/mL) displayed equal rates of annexin V positivity as non-rGd-eno treated cells (regions R2 and R4; **Figure 7Ba, b**). The propidium iodide staining (region R1) was also similar under both conditions (**Figure 7Ba, b**). However, annexin V was tightly bound to H2O2-treated IEC-6 cells (positive control for apoptosis), and these cells were also stained with propidium iodide (region R2) (**Figure 7Bc**). These results strongly suggest that apoptosis is not the principal mechanism of cell death triggered by rGd-eno.

**Figure 8:**
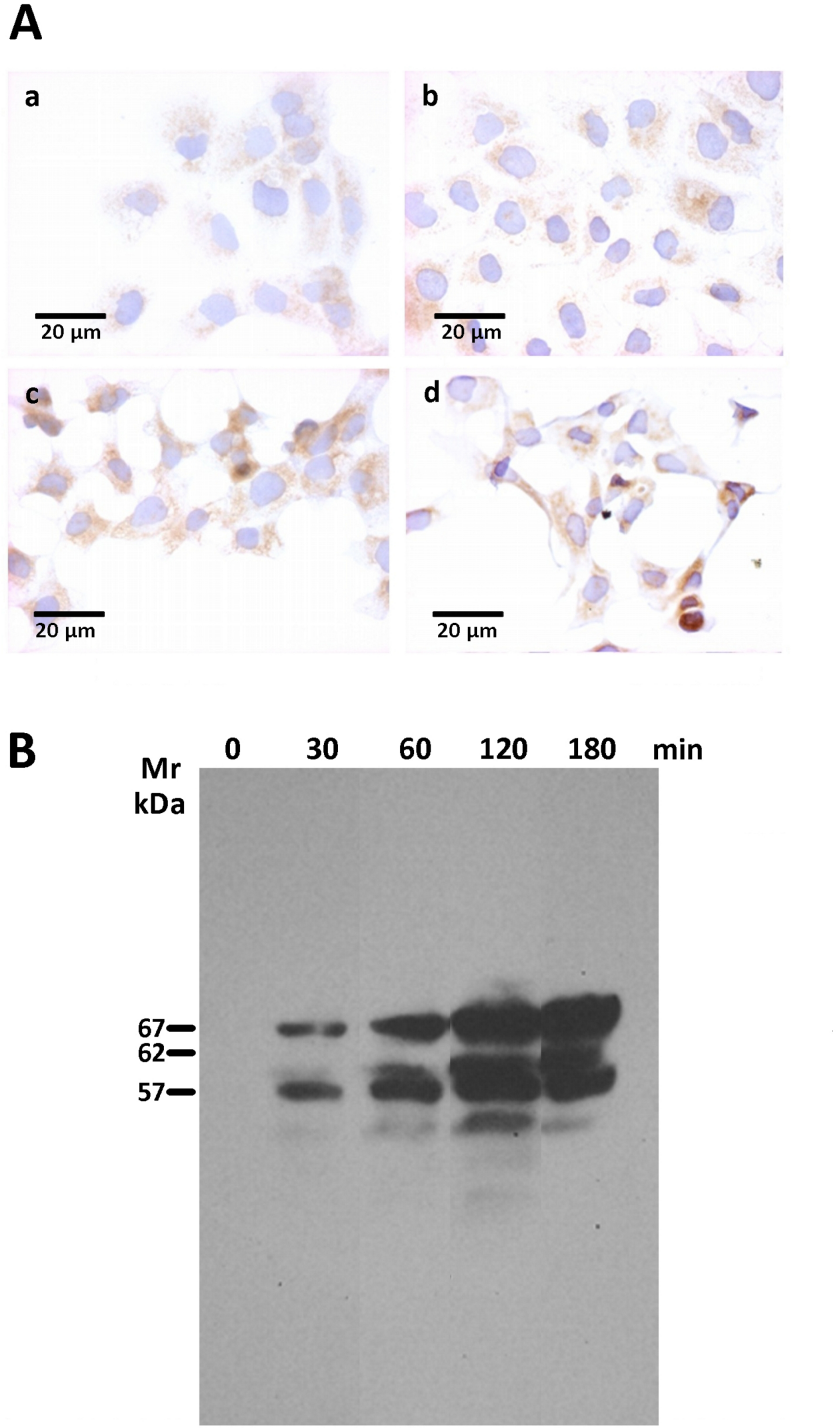
Determination of AIF activation upon the exposure of IEC-6 cells to rGd-eno. AIF activation was studied in IEC-6 cell monolayers exposed to rGd-eno (100 μg/mL; relative glycolytic activity 58 μmol/minxmg of protein) for 3h. Then, immunohistochemistry and Western blot analysis of AIF expression in IEC-6 cell lysates were performed. **(A)** Immunohistochemistry images of no treated cells **(a, b)** and treated cells with rGd-eno at **(c)** 90 min and **(d)** 120 min. **(B)** Expression of AIF in its 3 forms (precursor, mature and apoptogenic) at different times of exposure to rGd-eno (0 min, 30 min, 60 min, 120 min, and 180 min).

Several other cell-death effectors, which included caspase 8, BID and LC3B were also assessed by immunofluorescence and immunohistochemistry assays. However, none of those cell death effectors displayed a differential expression between rGd-eno treated and non-treated cells (**Supplementary Figure 4**). Nevertheless, as shown in **Figure 8Ac, d** we observed a clear increase in levels of the necroptosis marker AIF in IEC-6 monolayers rGd-eno-treated compared with non-treated IEC-6 monolayers (**Figure 8Aa, b**), suggesting that rGd-eno triggers necroptosis in IEC-6. The role of AIF-mediated necroptosis has been suggested previously (Delavallée et al., 2011). To analyze the role of AIF in this process, extracts from a temporal course of IEC6 cells exposed to purified Gd-eno were studied for AIF (**Figure 8B**); the results showed that rGd-eno treatment stimulates the release and activation of AIF in IEC-6.

**Figure 9:**
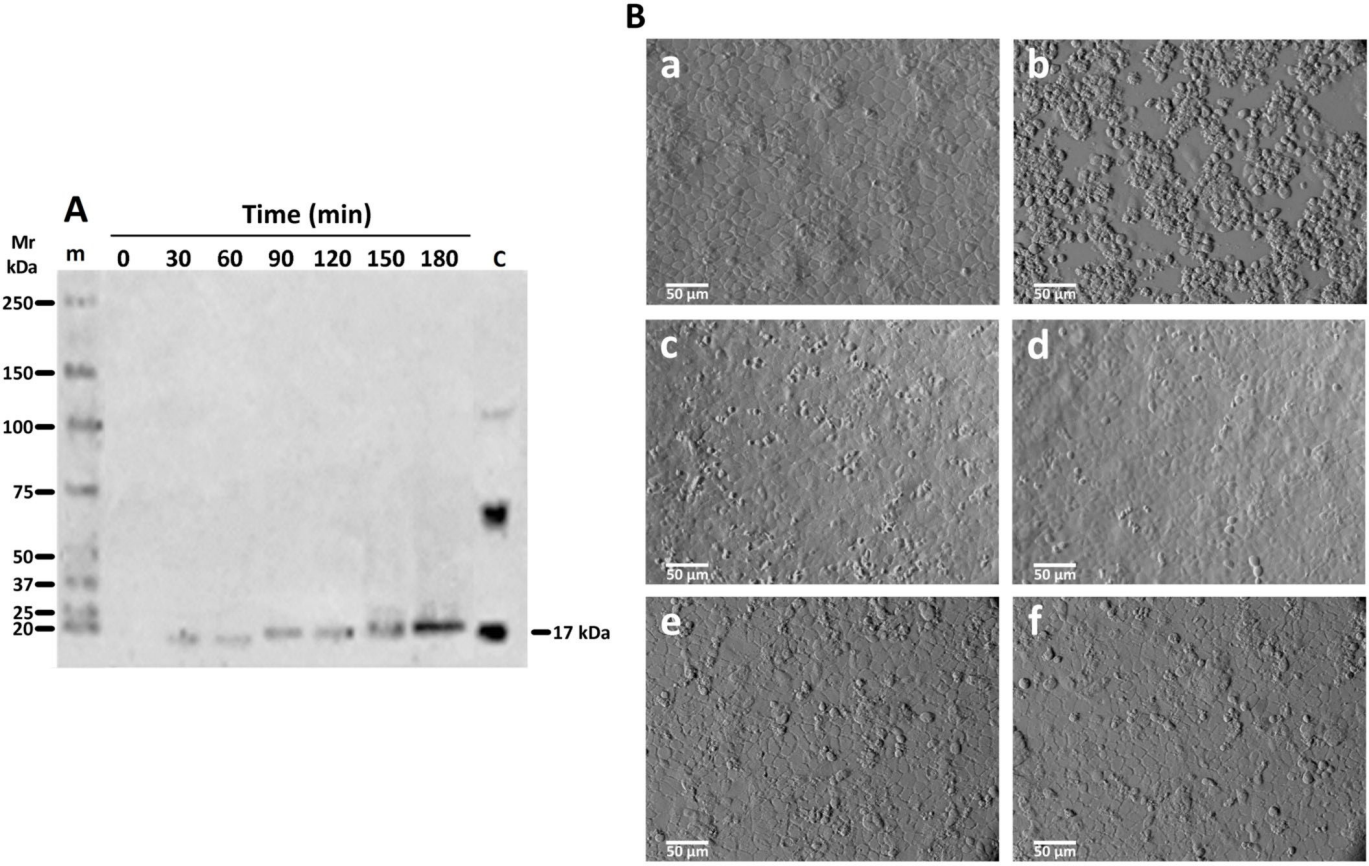
TNFα release in supernatants of IEC-6 cells exposed to rGd-eno and effect of the inhibition of TNFα activity with TAPI-0 and BAY 11-7082. Supernatants from IEC-6 cells incubated with rGd-eno at the indicated times were analyzed by Western Blot using anti-TNFα antibody. **(A)** Active TNFα, detected as a band of 17 kDa, Control **(C)** of non-active and active TNFα obtained from rat kidney extracts. **(B)** Inhibition of TNFα activation and IEC-6 cell damage by rGd-eno (100 μg/ml; relative glycolytic activity 58 μmol/min x mg of protein) was analyzed in the presence of different concentrations of TAPI-0 or BAY 11-7082. Untreated IEC-6 cells **(a)**, IEC-6 cells after 3 h treatment with rGd-eno **(b)** then with TAPI-0 at 50 μM **(c)** and 100 μM **(d)** or with BAY 11-7082 at 40 mM **(e)**, and 60 mM **(f)**. Micrographs are representative of three independent experiments.

Since AIF production is linked to TNFα signaling (Jurewicz et al., 2005; Xu et al., 2018), we evaluated the presence of this cytokine in supernatants from cultures of Giardia and IEC-6 interactions exposed to rGd-eno and in extracts of IEC-6 (only) (**Figure 9A**); rGd-eno treatment enhanced the presence of TNFα (17 kDa band) over time. Using a TNF-α processing inhibitor (TAPI-0) that blocks the generation of the active form of TNFα (17 kDa) by inhibiting TACE, we showed that TAPI-0 inhibited rGd-eno-induced damage to IEC-6 (**Figure 9Bc,d**). In addition, BAY 11-7082, a nuclear NF-kappa B inhibitor that reduces TNFα expression/secretion (Zhang and Feng, 2022) also decreased cell damage in a dose–dependent manner (**Figure 9b,e,f**). The indirect effect of BAY 11-7082 on the TNFα production/signaling could partially explain the reduction in rGd-eno-mediated damage in IEC-6 cell monolayers. Taken together, these results showed that the activation of AIF and TNFα release following the interaction of IEC-6 cell monolayers with rGd-eno is associated with necroptotic-like damage to epithelial cells.

## Discussion

Enolase, an enzyme of the glycolytic/gluconeogenic metabolism, has been detected in the culture supernatant of different microorganisms (Lamonica et al., 2005; DelVecchio et al., 2006; Chitlaru et al., 2007) suggesting that this enzyme has functional roles other than in carbohydrate metabolism (Moore, 2004). In the case of Giardia, enolase (Gd-eno) is released into the medium by trophozoites during its interaction with epithelial cells (Ringqvist et al., 2011; Ma’ayeh and Brook-Carter, 2012) in a monomeric form, as shown here. However, the exact mechanism of enolase secretion in Giardia, how the enzyme arrives at the cell surface and how it is bound to the cell membrane are still unclear. Interestingly, the protein neither possesses a predicted transmembrane region or glycosylphosphatidylinositol (GPI) anchor site nor a detectable N-terminal transit peptide, suggesting that it is not transported via the classical secretory pathway. Nevertheless, immunofluorescence assays using specific antibodies to rGd-eno revealed that this protein is present in the cytoplasm in small vesicles that could be part of the mechanism of secretion, as has been reported in Trypanosoma (Avilán et al., 2011), but this process needs further exploration.

Additionally, we demonstrated that Gd-eno is a protein with moonlighting functions, as observed for other parasites, and plays a role as an intracellular metabolic enzyme as well as an extracellular ligand for plasminogen. As a result of this interaction, plasmin activity is enhanced. Since enolase does not possess an intrinsic protease activity or protease domains, the generation of active plasmin with monomeric Gd-eno is possibly due to an induction of conformational changes that allow the exposure of zymogen domains in the plasminogen following ligand binding. Regarding Gd-eno-HsPlgII interactions, these were previously hypothesized and analyzed in silico, in terms of the “open” (with one Mg2+ bound, here named as partially active) and “closed” (with two Mg2+ ions bound, named here as “fully active”) conformations of the Gd-eno dimer (Aguayo-Ortiz et al., 2017). The importance of the PID and K266, along with the modeling data help to predict the putative conformational changes occurring in the Gd-eno dimer following Mg2+ binding (Aguayo-Ortiz et al., 2017). However, our findings suggest that the secreted Gd-eno monomer can interact with host plasminogen at its SP, K3 and K4 domains and the lysine-rich interactive environment, as suggested by the inhibition of plasmin activity with the lysine analog ε-ACA. In addition, the docking of the closed or fully active conformation of Gd-eno monomer suggests that Gd-eno might interact with an open conformation of HsPlg and might be required to promote optimal plasmin activity. Once plasmin is generated and activated, it can cleave fibrin, fibronectin and laminin (Plow et al., 1995), and activates other proteolytic enzymes, resulting in the cleavage of collagen, elastin, proteoglycans (Murphy et al., 1999) and intercellular junctions (Attali et al., 2008), or induces the release of cytokines – as evidenced here. This process could, therefore, be responsible for inducing the damage in epithelial cells before undergoing cell death.

A key finding here was the release of the active form of TNFα (17 kDa) into the medium following the incubation of rGd-eno with IEC-6 epithelial cells. The damage induced by rGd-eno on the cells was inhibited using inhibitors of TNFα activators, such as TAPI-0, indicating that the TNFα released by epithelial cells after the interaction of enolase plays a role in the process. Therefore, is easy to speculate that the stimulation and enhancing of the plasminogen-plasmin system could mediate the activation of TACE (DasGupta et al., 2009). Furthermore, the reduction of epithelial cell damage in the monolayers incubated with rGd-eno after the inhibition of TNFα downstream signaling with BAY 11-7082, confirms that TNFα signaling participates in the epithelial cell damage, triggered by rGd-eno. Such findings correlate with previous reports linking necroptosis to the presence of TNFα (Günther et al., 2011). In addition, the increase activation of AIF in IEC-6 cells by rGd-eno supports the idea that rGd-eno induces a necroptosis-like damage in epithelial cells via TNFα. Currently, several studies have shown that secretion of TNFα was indeed stimulated during giardiasis (Saghaug et al., 2016), or induced by parasite components (Muñoz-Cruz et al., 2010). Indeed, direct activation of mast cells by live G. duodenalis trophozoites or trophozoite-derived antigens induced the release of TNF-α from mast cells through an Ig-independent pathway (Muñoz-Cruz et al., 2010). In a more recent study (Muñoz-Cruz et al., 2018), a parasite extract-fraction, named F2, which contains among other proteins, ADI and enolase, induced the release of IL-6 and TNF-α by mast cells, which have been shown previously to play an important role in controlling infection with both. G. muris and G. duodenalis (Fink and Singer, 2017). Interestingly, the analysis of the mitochondrial flavoprotein AIF, a caspase-independent death effector (Candé et al., 2002), revealed its possible role in the epithelial damage triggered by the enolase/TNFα mechanism. Immunohistochemistry and Western blot analyses showed an increased activation of AIF into its three principal forms: precursor, mature and apoptogenic. In conjunction, these results also support the notion that rGd-eno induces a necroptosis-like damage in IEC-6 monolayers in a way that occurs downstream of TNFα.

The pathway triggering the necroptotic-like damage caused by rGd-eno in epithelial cells is presently unknown and no microbial enolase has yet been reported to induce necroptosis of host cells. Based on the experimental findings here, it is plausible that rGd-eno interacts with HsPlg, to generate plasmin activity that degrade extracellular matrix components, and promotes enterocyte cell death. In this context, Gd-eno was detected as a monomer when secreted by trophozoites and, therefore, the presence of a monomeric Gd-eno may be necessary to trigger necroptotic-like damage in epithelial cell monolayers. In this context, the previously hypothesized monomer state of enolases could explain, at least in part, its multifunctionality (Pal-Bhowmick et al., 2007). Firstly, enolase has been reported previously to function as a plasminogen receptor for bacterial, fungal, protozoal and helminth pathogens that modulates the innate immunity and promotes the damage of host tissues as well as the disturbance of the fibrinolytic system, thereby facilitating pathogen invasion and establishment (Ayón-Núñez et al., 2018). Secondly, the secretion of Gd-eno monomer was enhanced upon interaction of trophozoites with epithelial cells, in agreement with previous reports, demonstrating the increase of both Gd-eno mRNA levels and Gd-eno protein amounts in supernatants due to these interactions (Ringqvist et al., 2008). Thirdly, the monomeric nature of secreted Gd-eno correlates not only with the experimental data obtained here and with the catalytic ability of the endogenous enzyme but also allowed to determine in a functional context its plasminogen-activating role and its driving contribution in the necroptosis-like damage of epithelial cells.

Lastly, to oversee the epithelial cell damage induced by the enolase we used high concentrations of the enzyme (100 μg/mL) to exacerbate the outcome. However, ex vivo the trophozoites can locally induce cell damage in the enterocytes that are present in the surface of the villi and the injuries strongly mimic the observed in our in vitro model. Thus, based on the results we can envision two scenarios that contribute to the pathogenesis of the parasite: a) in a local microenvironment, the concentration of secreted enolase is higher than expected and, b) the parasite displays tropism for areas enriched with enolase receptor and that promotes the enolase-receptor interactions. However, with our results, the presence of additional mechanisms enolase-dependent that could be important in the pathogenesis induced by Giardia cannot be ruled out. We consider that all concepts are interesting and must be investigated in the future.

In conclusion, the present findings support the role of G. duodenalis enolase in the damage of epithelial cells; therefore, this enzyme should be considered as an element of the virulence factors in this parasite (Argüello-García and Ortega-Pierres, 2021). Future studies of the participation of enolase during the interaction of Giardia trophozoites with host intestinal epithelial cells using experimental animal models will provide insights into its role on the pathogenesis of giardiasis.

## Data availability statement

The original contributions presented in the study are included in the article/**Supplementary Material**, further inquiries can be directed to the corresponding author/s.

## Author contributions

GO-P: conceptualization and supervision. EB-E RF-L, RA-G, RM-R and RB-C: experiments. GO-P, EB-E and RA-G: writing of the original draft. N-P writing, comments and revision. GO-P funding acquisition. All authors contributed to the article and approved the submitted version.

## Funding

This work was supported in part by Fondo Sectorial Secretaría de Educación Pública-Consejo Nacional de Ciencia y Tecnología (SEP–CONACYT) México, Grant number A1-S-39422 and by the Miguel Aleman Foundation and Cinvestav. México.

## Acknowledgments

The authors wish to express special thanks to Rusely Encalada (INCar, Mexico) for her help in determining enolase activity and to Emma Saavedra (INCar, Mexico) for her valuable comments and suggestions while performing this work. We thank Silvia Espinosa-Matías (UNAM Mexico) and Bibiana Chávez Munguía (Cinvestav Mexico) for their skillful assistance in obtaining scanning electron microscopy micrographs and Sara Huerta Yépez (HIMFG Mexico) for her advice and help in the detection of cell death markers. We also thank María Luisa Bazán Tejeda and Antonio Sandoval Cabrera (Cinvestav Mexico) for technical support in cloning Giardia enolase, to Adrián Chávez Cano and Arturo Pérez-Taylor (Cinvestav Mexico) for the artwork. We are most grateful to Prof. Robin B. Gasser for editorial comments on the manuscript. We thank Prof. Keith Gull, University of Oxford, Oxford, UK for the donation of the anti- α-tubulin antibody and to Héctor Romero (Cinvestav Mexico) for providing antibodies against TNFα.

## Conflict of interest

The authors declare that the research was conducted in the absence of any commercial or financial relationships that could be construed as a potential conflict of interest.

## Publisher’s note

All claims expressed in this article are solely those of the authors and do not necessarily represent those of their affiliated organizations, or those of the publisher, the editors and the reviewers. Any product that may be evaluated in this article, or claim that may be made by its manufacturer, is not guaranteed or endorsed by the publisher.

## Supplementary material

Supplementary Figure 1 Alignment of the amino-acid sequences of Giardia enolase with similar proteins from different organisms. Amino-acid sequence alignment of enolases from Homo sapiens (a-enolase, accession number NP_001419), Saccharomyces cerevisiae (accession number AAA88713.1), Trypanosoma brucei (accession number XP_822542) and G. duodenalis assemblage A (GL50803_11118). Cyano-shaded letters indicate residues involved in ligand (phosphoenolpyruvate) binding and residues involved in Mg2+ binding are shown in brown-shaded letters. Green-shaded letters are unique reactive active-site residues found in enolases from some protozoa (Avilán et al., 2011) including Giardia. The three lysines considered as primary plasminogen binding sites are enclosed in green box whilst the red box indicates the proposed plasminogen binding motif. The underline in red identifies a conserved loop involved in the protonation of 2-phosphaglycerate by H159. The enolase signature is indicated in bold letters. Residues in red are that forming ionic bound in the enolase dimer. Yellow-shaded letters are variant residues between Giardia A, B and E assemblages and black-shaded letters are residues absent in assemblage E. Multiple sequence alignment was carried out using the CLUSTALW 2.1 software (http://www.genome.jp/tools/clustalw/) with default parameters

Supplementary Figure 2 Enolase gene amplification, cloning and production of recombinant enolase. **(A)** Plasmid map for PET-100_ENO with the enolase gene in the PET-100 D-TOPO® (Invitrogen) transition-expression vector and 6XHis Tag. **(B)** PCR amplification of a 1500 bp product resolved in a 1% agarose gel and stained with ethidium bromide which was, confirmed to be the enolase gene by automatic DNA sequencing. **(C)** SDS-PAGE of the fractions obtained during the purification process of rGd-eno; demonstrate an enriched band of ≈48 kDa corresponding to enolase obtained in the faction C.

Supplementary Figure 3 Absence of damage in IEC-6 cells by extracts from E. coli BL21 Star lacking Gd-eno. IEC-6 monolayers were incubated for 2h at 370C in 2 cm2 wells in serum free DMEM medium **(A)** Control IEC-6 cell monolayers incubated with DMEM only, showing the normal morphology of the cell monolayer. **(B)** IEC-6 monolayers incubated with E. coli BL21 Star extracts dialyzed and passed through a High Capacity Endotoxin Removal (Pierce™) column. **(C)** IEC-6 cell monolayers exposed to rGd-eno.

Supplementary Figure 4 Determination of IEC-6 monolayer cell confluence on cell damage induced by rGd-eno. IEC-6 cell monolayers were incubated with rGdeno as described in material and methods. IEC-6 cell monolayers were grown at three different confluences **(D)** 50%, **(E)** 70% and **(F)** 100% in the presence of 100 mg/ml; of rGd-eno. (relative glycolytic activity 58 mmol/ minx mg of protein). Untreated IEC-6 cell monolayers grown at the same confluences of 50% **(A)**, 70% **(B)** and 100% **(C)** were used as negative controls. Micrographsare representative of three independent experiments.

Supplementary Figure 5 Determination of Caspase 3, Caspase 8, Caspase 9, BID and LC3B activity. Apoptosis and autophagy were analyzed by immunofluorescence **(A)**. IEC-6 cell nuclei were stained with DAPI **(A, B)**while, caspase 3 **(C)** and LC3B **(D)** are shown in green. Immunohistochemistry assays with IEC-6 cells monolayers incubated in the absence **(A–C)** or in the presence of rGd-eno **(D–F)**. Caspase 3 **(A, D)**, Caspase 8 **(B, E)** or BID **(C, F)** was performed. Scale bar = 20mm.
