# Supplementary figures and images for: Giardia duodenalis enolase is secreted as monomer during trophozoite-epithelial cell interactions, activates plasminogen and induces necroptotic damage
Source: Front Cell Infect Microbiol. 2022 Aug 25;12:928687. doi: 10.3389/fcimb.2022.928687 (PMC9452966; doi:10.3389/fcimb.2022.928687)

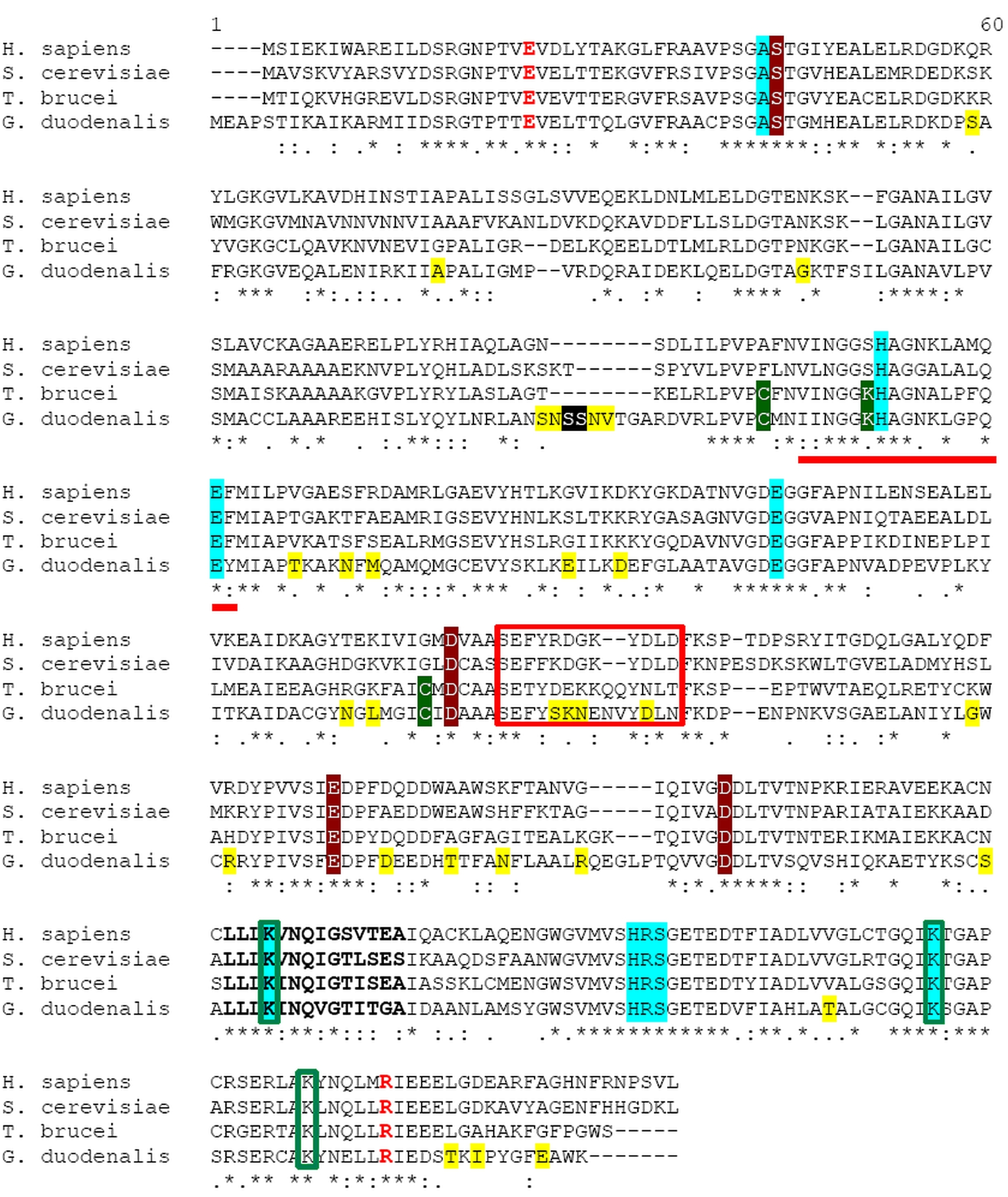

Supplement: Supplementary Figure 1 — Alignment of the amino-acid sequences of Giardia enolase with similar proteins from different organisms. Amino-acid sequence alignment of enolases from Homo sapiens (a-enolase, accession number NP_001419), Saccharomyces cerevisiae (accession number AAA88713.1), Trypanosoma brucei (accession number XP_822542) and G. duodenalis assemblage A (GL50803_11118). Cyano-shaded letters indicate residues involved in ligand (phosphoenolpyruvate) binding and residues involved in Mg2+ binding are shown in brown-shaded letters. Green-shaded letters are unique reactive active-site residues found in enolases from some protozoa (Avilán et al., 2011) including Giardia. The three lysines considered as primary plasminogen binding sites are enclosed in green box whilst the red box indicates the proposed plasminogen binding motif. The underline in red identifies a conserved loop involved in the protonation of 2-phosphaglycerate by H159. The enolase signature is indicated in bold letters. Residues in red are that forming ionic bound in the enolase dimer. Yellow-shaded letters are variant residues between Giardia A, B and E assemblages and black-shaded letters are residues absent in assemblage E. Multiple sequence alignment was carried out using the CLUSTALW 2.1 software (http://www.genome.jp/tools/clustalw/) with default parameters [file Image_1.jpeg]

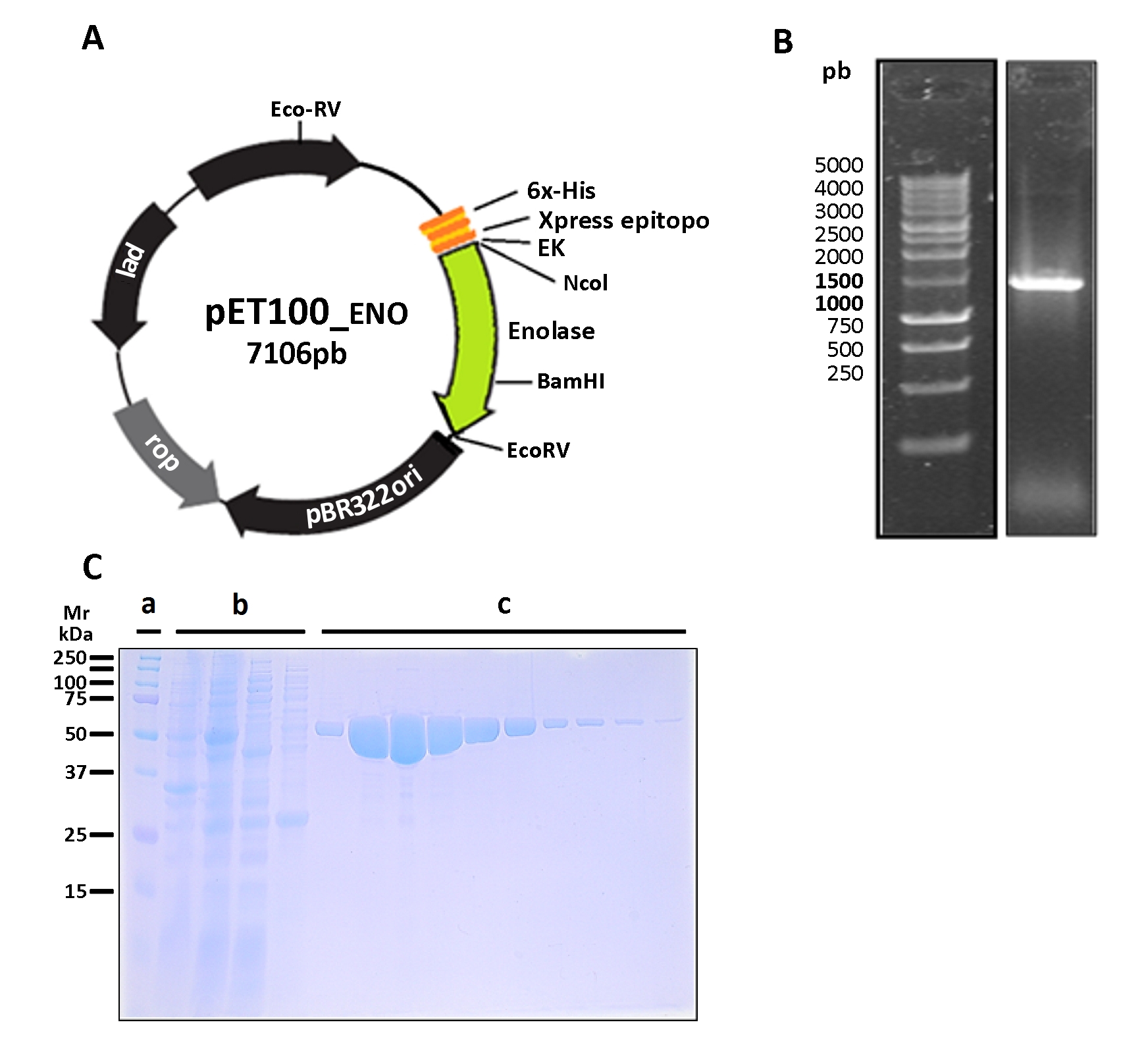

Supplement: Supplementary Figure 2 — Enolase gene amplification, cloning and production of recombinant enolase. (A) Plasmid map for PET-100_ENO with the enolase gene in the PET-100 D-TOPO® (Invitrogen) transition-expression vector and 6XHis Tag. (B) PCR amplification of a 1500 bp product resolved in a 1% agarose gel and stained with ethidium bromide which was, confirmed to be the enolase gene by automatic DNA sequencing. (C) SDS-PAGE of the fractions obtained during the purification process of rGd-eno; demonstrate an enriched band of ≈48 kDa corresponding to enolase obtained in the faction C. [file Image_2.jpeg]

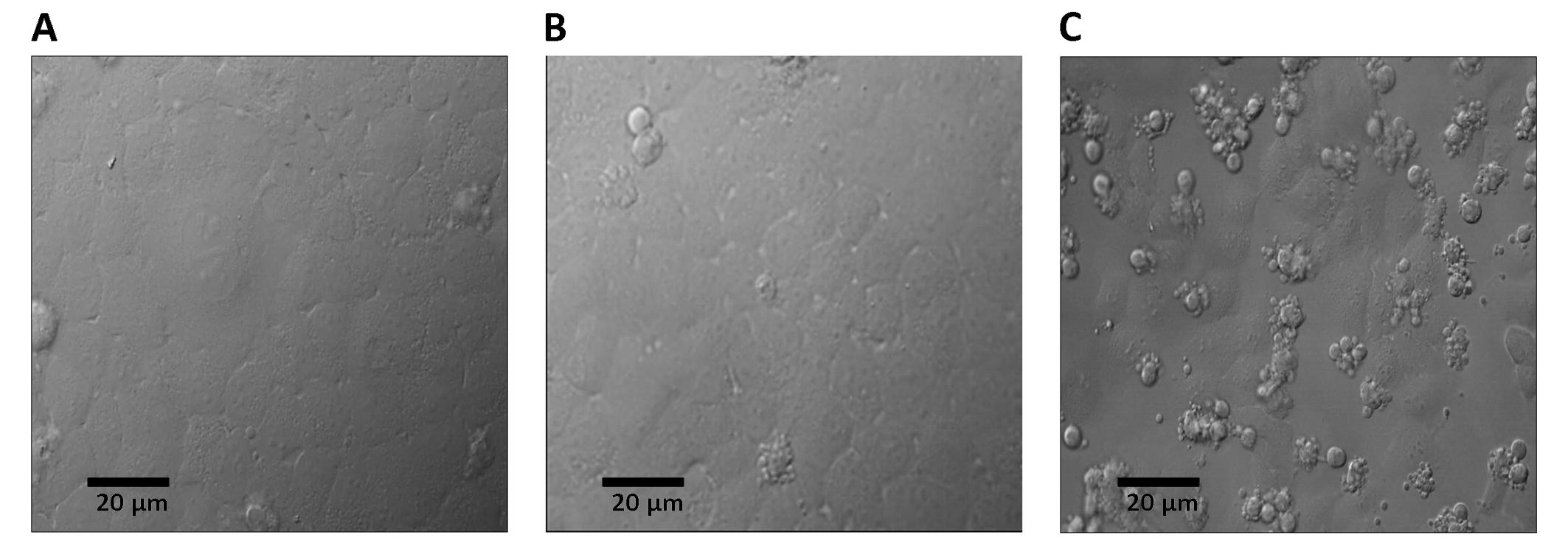

Supplement: Supplementary Figure 3 — Absence of damage in IEC-6 cells by extracts from E. coli BL21 Star lacking Gd-eno. IEC-6 monolayers were incubated for 2h at 370C in 2 cm2 wells in serum free DMEM medium (A) Control IEC-6 cell monolayers incubated with DMEM only, showing the normal morphology of the cell monolayer. (B) IEC-6 monolayers incubated with E. coli BL21 Star extracts dialyzed and passed through a High Capacity Endotoxin Removal (Pierce™) column. (C) IEC-6 cell monolayers exposed to rGd-eno. [file Image_3.jpeg]

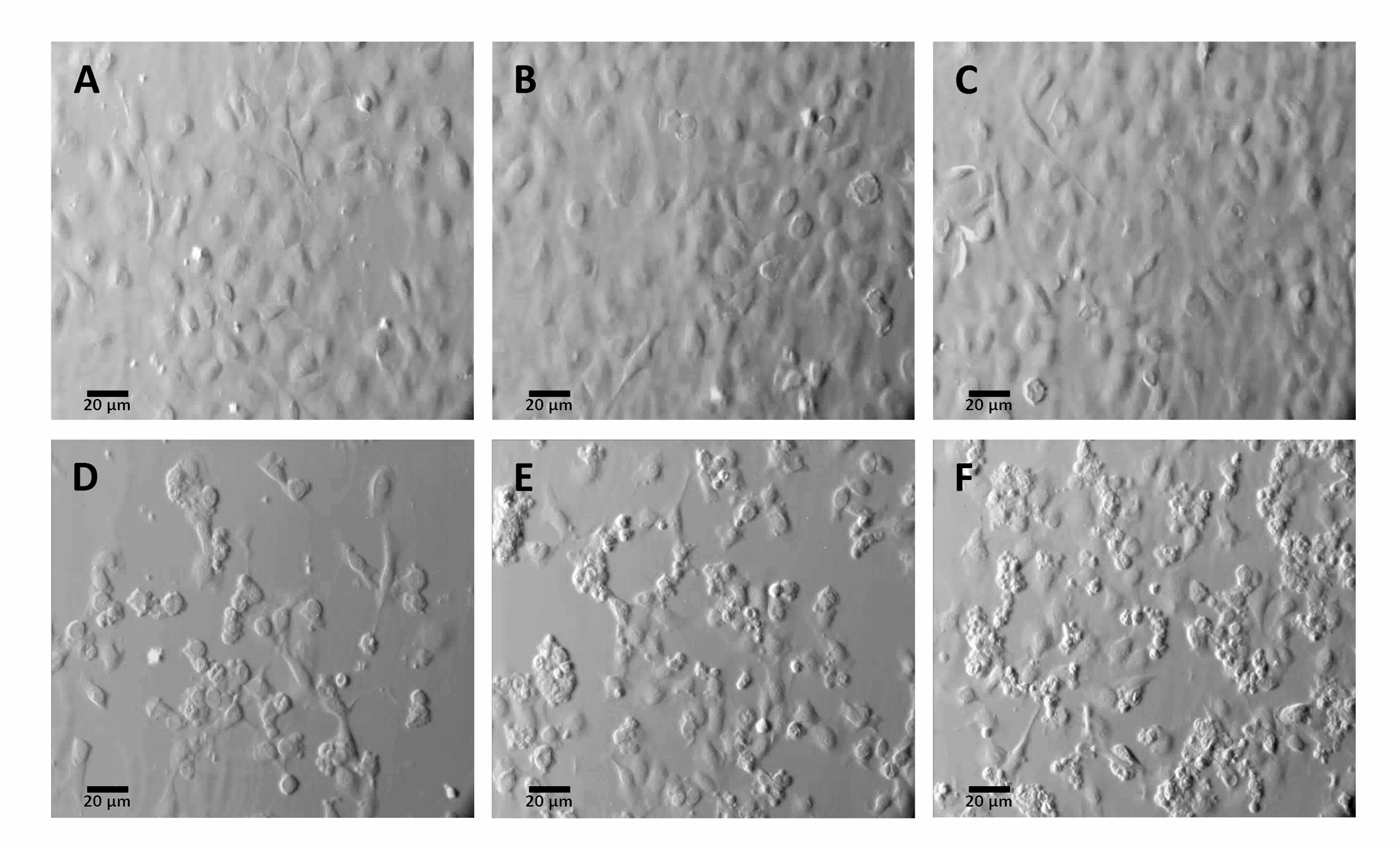

Supplement: Supplementary Figure 4 — Determination of IEC-6 monolayer cell confluence on cell damage induced by rGd-eno. IEC-6 cell monolayers were incubated with rGdeno as described in material and methods. IEC-6 cell monolayers were grown at three different confluences (D) 50%, (E) 70% and (F) 100% in the presence of 100 mg/ml; of rGd-eno. (relative glycolytic activity 58 mmol/ minx mg of protein). Untreated IEC-6 cell monolayers grown at the same confluences of 50% (A), 70% (B) and 100% (C) were used as negative controls. Micrographsare representative of three independent experiments. [file Image_4.jpeg]

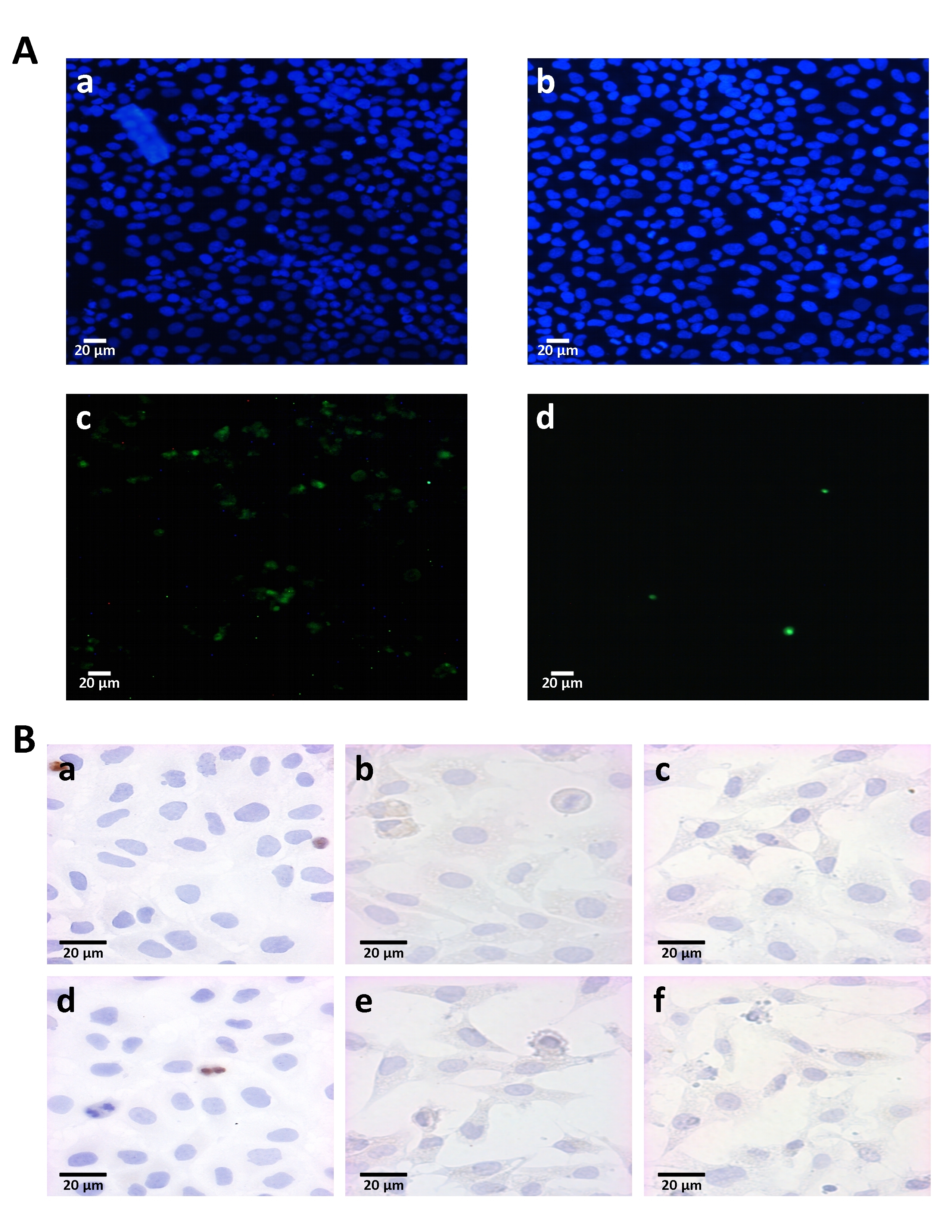

Supplement: Supplementary Figure 5 — Determination of Caspase 3, Caspase 8, Caspase 9, BID and LC3B activity. Apoptosis and autophagy were analyzed by immunofluorescence (A). IEC-6 cell nuclei were stained with DAPI (A, B)while, caspase 3 (C) and LC3B (D) are shown in green. Immunohistochemistry assays with IEC-6 cells monolayers incubated in the absence (A–C) or in the presence of rGd-eno (D–F). Caspase 3 (A, D), Caspase 8 (B, E) or BID (C, F) was performed. Scale bar = 20mm. [file Image_5.jpeg]
